# Impact of pasteurization on the self-assembly of human milk lipids during digestion

**DOI:** 10.1016/j.jlr.2022.100183

**Published:** 2022-02-16

**Authors:** Syaza Y. Binte Abu Bakar, Malinda Salim, Andrew J. Clulow, Adrian Hawley, Joseph Pelle, Donna T. Geddes, Kevin R. Nicholas, Ben J. Boyd

**Affiliations:** 1 Drug Delivery, Disposition and Dynamics, Monash Institute of Pharmaceutical Sciences, Monash University, Parkville, Victoria, Australia; 2 Australian Synchrotron, ANSTO, Clayton, Victoria, Australia; 3 School of Molecular Science, The University of Western Australia, Crawley, Western Australia, Australia; 4 Department of Pharmacy, University of Copenhagen, København Ø, Denmark

**Keywords:** human breast milk, lipids, digestion, development, nutrition, calcium levels, colloidal structures, bile salts, fatty acid, gas chromatography, BSSL, bile salt-stimulated lipase, FAME, FA methyl ester, GC-FID, GC coupled to a flame ionization detector, GIT, gastrointestinal tract, MAG, monoacylglycerol, PC, principal component, PCA, principal component analysis, PTV, programmed temperature vaporization, SAXS, small-angle X-ray scattering, TAG, triacylglycerol

## Abstract

Human milk is critical for the survival and development of infants. This source of nutrition contains components that protect against infections while stimulating immune maturation. In cases where the mother's own milk is unavailable, pasteurized donor milk is the preferred option. Although pasteurization has been shown to have minimal impact on the lipid and FA composition before digestion, no correlation has been made between the impact of pasteurization on the FFA composition and the self-assembly of lipids during digestion, which could act as delivery mechanisms for poorly water-soluble components. Pooled nonpasteurized and pasteurized human milk from a single donor was used in this study. The evolving FFA composition during digestion was determined using GC coupled to a flame ionization detector. In vitro digestion coupled to small-angle X-ray scattering was utilized to investigate the influence of different calcium levels, fat content, and the presence of bile salts on the extent of digestion and structural behavior of human milk lipids. Almost complete digestion was achieved when bile salts were added to the systems containing high calcium to milk fat ratio, with similar structural behavior of lipids during digestion of both types of human milk being apparent. In contrast, differences in the colloidal structures were formed during digestion in the absence of bile salt because of a greater amount of FFAs being released from the nonpasteurized than pasteurized milks. This difference in FFAs released from both types of human milk could result in varying nutritional implications for infants.

## Graphical abstract

**Figure:**
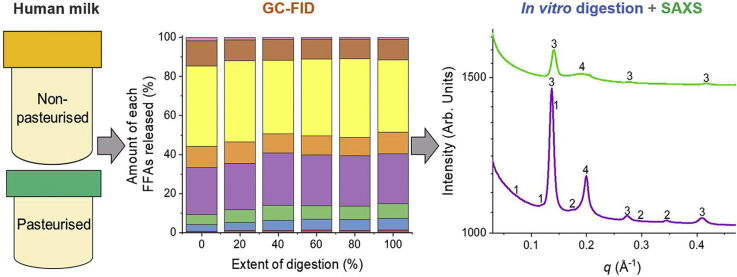

Breastfeeding in the first 6 months of life is associated with the healthy growth and development of infants (1). Human milk is universally accepted as the normative standard for infant feeding because of its unique nutritional composition and the presence of bioactive components that lower the risk of illnesses while stimulating immune function, establishing the gut microbiome, signaling physiological development, and providing antimicrobial activity to reduce infection (2). It has been shown that the mother's own milk offers protection against diseases, such as diarrhea, necrotizing enterocolitis, and respiratory tract infections (3, 4, 5). However, in cases where the mother's own milk is unavailable, pasteurized donor human milk is the preferred option (6). This is often the case for hospitalized preterm or ill infants receiving care in neonatal intensive care units. To ensure that pasteurized donor milk is free from viral and bacterial pathogens that might be either transmitted from the donor or acquired during collection and storage of milk, donor human milk is subjected to a holder pasteurization process (62.5°C for 30 min) (7). Although pasteurization ensures the microbiological safety of the milk, this heat treatment process might inactivate enzymes such as bile salt-stimulated lipase (BSSL), which is important in the lipid digestion process (8). Hence, intestinal lipid absorption could be significantly altered when an infant is fed with pasteurized instead of nonpasteurized human milk (9).

Digestion of lipids is a crucial process in enabling absorption of the polar digestion products to occur. The generation of more polar lipids induces the formation of lyotropic lipid liquid crystalline structures (10), which are believed to play a critical role in promoting the transport and bioavailability of poorly water-soluble elements to the systemic circulation. The total lipid content of milk comprises around 98 wt% triacylglycerols (TAGs), but the total lipid content and FA composition in human milk varies with diet, age, and parity (11). TAGs are molecules with a glycerol backbone esterified with FAs at the *sn*-1, *sn*-2, and *sn*-3 positions (12). Human milk fat is characterized by high contents of palmitic (C16:0) and oleic acids (C18:1), with the former primarily esterified at the *sn*-2 position and the latter concentrated in the *sn*-1 and *sn*-3 positions. The selective distribution of these FAs in the TAG molecules contributes to the overall enhancement of digestion and absorption of lipids in the gastrointestinal tract (GIT) (13, 14). Through the process of lipolysis, TAGs are hydrolyzed into diacylglycerols, monoacylglycerols (MAGs), and FFAs. This results in approximately 95–98% of lipid absorption in the small intestine (15). Pancreatic lipase in the upper small intestine accounts for more than 70% of the lipid digestion by hydrolyzing the two outer ester bonds leading to the liberation of one *sn*-2 MAG and two FFAs (16). While this occurs, gall bladder contractions lead to the secretion of bile, containing phospholipids, cholesterol, and bile salts (17). These bile salts further help to stabilize the milk lipid droplets and subsequently integrate the insoluble products from the hydrolysis of TAGs to form vesicles, mixed micelles, and more complex lipid liquid crystalline systems, thus increasing the oil-water surface area for hydrolytic activity.

BSSL, an enzyme constituent of raw human milk, in part enables the self-assembly process through the release of FFAs (18). BSSL has a higher intestinal activity than pancreatic lipase in hydrolyzing ester bonds and is more effective at liberating the long-chain polyunsaturated FAs that are essential to the development of an infant's organs such as the brain and central nervous system (19). Thus, although infants have low intraluminal bile salt concentrations (1–5 mM), the presence of BSSL can drive the digestion of lipids in the case where pancreatic lipase is insufficient to do so. In addition to bile salts, endogenous calcium, which is naturally found in milk and typically included in the digestion buffers used in in vitro digestion experiments, also increases the extent of digestion through the removal of the FAs from the oil-water interface of the lipid droplet by calcium ions (20). As this occurs, not only is the accessibility of the substrate for the lipase increased but the interaction between the calcium ions and FAs also results in the formation of calcium soaps. Hence, both bile salts and calcium act as enhancement tools in the digestion process, which could affect the fate of lipophilic nutrients in the systemic circulation.

Currently, the majority of the published studies either focus on the lipid composition of human milk before digestion or evaluate the self-assembly of lipids during digestion using in vitro models (14, 21, 22). However, there have been limited reports that investigate the correlation between these two aspects. This study illustrates the impact of pasteurization on the FFA composition of human milk and the subsequent effect on the types of lipid liquid crystalline phases formed during in vitro digestion (Fig. 1). GC coupled to a flame ionization detector (GC-FID) was used to quantify the amount of FFAs released during digestion. Based on these results, the influence on the FFAs released during digestion on the formation of colloidal structures was determined using small-angle X-ray scattering (SAXS).

**Fig. 1:**
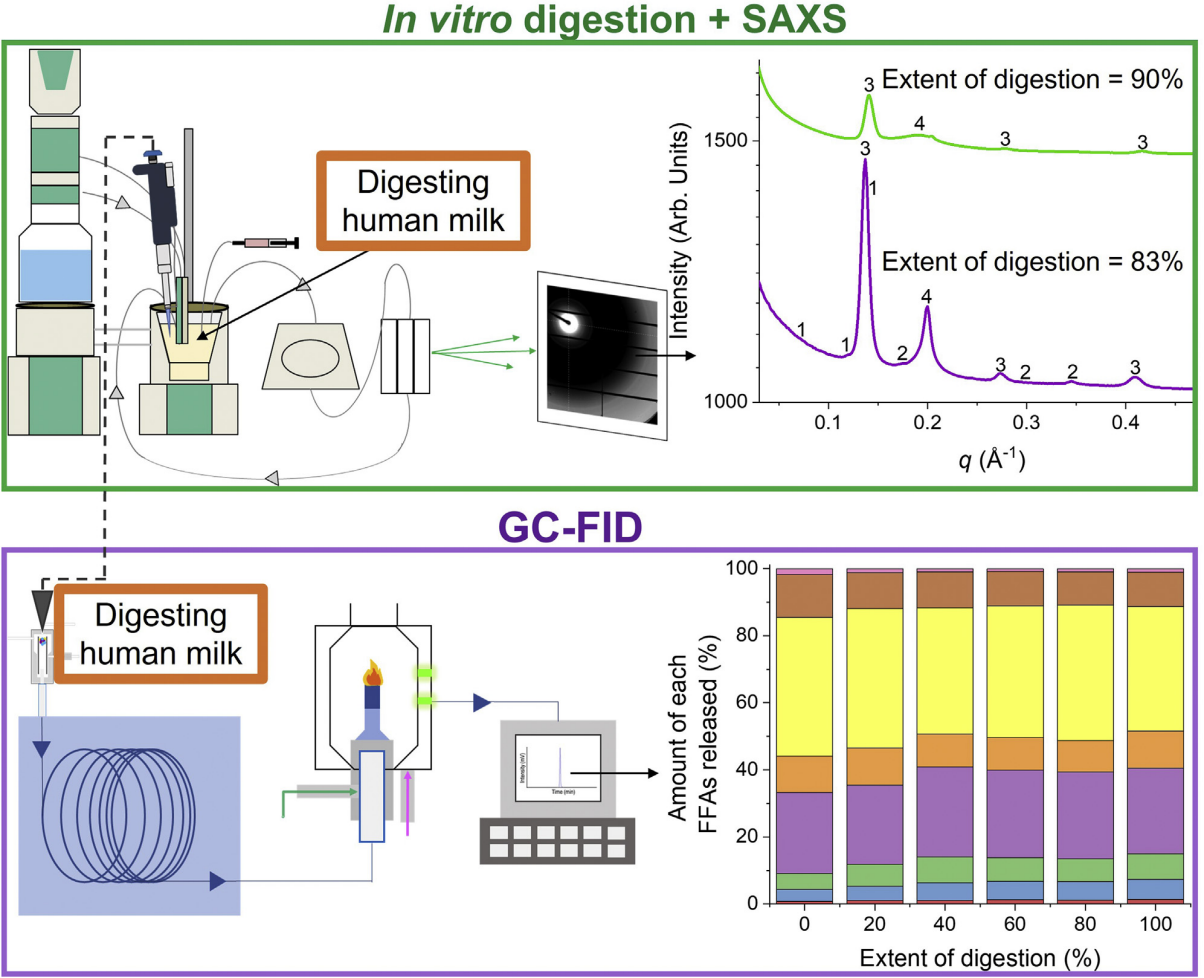
Schematic diagram illustrating the two primary experimental approaches to link the FFA composition of nonpasteurized and pasteurized human milk determined using GC coupled to a flame ionization detector (GC-FID) and self-assembly of lipids monitored using an in vitro lipolysis model coupled to in situ small-angle X-ray scattering (SAXS) during the digestion of human milk.

## Materials and methods

### Standards and materials

Human breast milk was donated by the Mercy Health Breastmilk Bank (Heidelberg, Victoria, Australia) with ethics approval from the Mercy Health Human Ethics Research Committee (application 2017-035) and following the principles of the Declaration of Helsinki. The human milk samples were stored at −20°C, and prior to analyses, they were thawed and agitated using a vortex mixer for 5 min. Tricaprylin (C8:0, >99% purity), methyl undecenate (C11:1, >99% purity), glyceryl tridecanoate (C13:0, >99% purity), glyceryl triundecanoate (C11:0, >99% purity), monopalmitin (C16:0, >99% purity), and oleic acid (C18:1, >99% purity) were obtained from Nu-Chek Prep (Elysian, MN). FA methyl ester (FAME) standard mixtures containing the following methyl esters were also purchased from Nu-Chek Prep (Elysian, MN): methyl butyrate (C4:0), methyl pentanoate (C5:0), methyl hexanoate (C6:0), methyl heptanoate (C7:0), methyl octanoate (C8:0), methyl nonanoate (C9:0), methyl decanoate (C10:0), methyl undecanoate (C11:0), methyl laurate (C12:0), methyl tridecanoate (C13:0), methyl myristate (C14:0), methyl pentadecanoate (C15:0), methyl palmitate (C16:0), methyl heptadecanoate (C17:0), methyl stearate (C18:0), methyl oleate (C18:1), methyl linoleate (C18:2), and methyl α-linolenate (C18:3). Tricaprin (C10:0, >98% purity), trilaurin (C12:0, >98% purity), trimyristin (C14:0, >95% purity), tristearin (C18:0, >80% purity), and triolein (C18:1, >80% purity with the major impurity being trilinolein) were purchased from Tokyo Chemical Industry Co (Tokyo, Japan). Calcium chloride dihydrate (>99% purity) and sodium hydroxide pellets (>97% purity) were purchased from Ajax Fine Chemicals (Seven Hills, New South Wales, Australia). Absolute ethanol was purchased from Merck (Darmstadt, Germany). 1,2-Dioleoyl-*sn*-glycero-3-phosphocholine was purchased from Cayman Chemical Company (Ann Arbor, MI). Hydrochloric acid (36% aqueous solution) was obtained from LabServ (Longford, Ireland). Sodium chloride (>99.7% purity) was purchased from Chem Supply (Gillman, SA, Australia). Tripalmitin (C16:0, ≥85% purity), sodium azide (>99% purity), chloroform (ACS, ISO, reagent), and methanol (HPLC basic) were obtained from Merck (Darmstadt, Germany). Methanolic HCl (3 M), *n*-hexane (≥98% purity, GC), tert-butyl methyl ether (99.8% purity, anhydrous), acetic acid (99.8% purity, anhydrous), 4-bromophenyl boronic acid, sodium taurodeoxycholate hydrate (95% purity), Trizma®-maleate (reagent grade), and pepsin from porcine gastric mucosa (lyophilized powder) were purchased from Sigma Aldrich (St Louis, MO). Pancreatin extract (US Pharmacopeia grade) was obtained from MP Biomedicals (Solon, OH). Fungal lipase 8000 was purchased from Connell Bros Australasia Pty Ltd (Croydon South, Victoria, Australia). Unless otherwise stated, all chemicals were used without further purification, and water was acquired from a Merck Q-POD Ultrapure Water Remote Dispenser (Darmstadt, Germany).

### Triglyceride composition analysis using GC-FID

#### Extraction of lipids

Total lipids were extracted from pooled nonpasteurized and pasteurized human milk samples by a single donor (n = 3 each) following the method previously described by Folch *et al.* (23). Briefly, human milk samples (1.61 ml each) were spiked with 2 mg/ml triundecanoin in chloroform as an internal standard. The samples were subsequently mixed with 1.08 ml chloroform and 0.54 ml methanol (2:1, v/v), agitated using vortex mixing, and centrifuged at 1,503 *g* for 10 min. The clear homogenate was then transferred to a separating funnel. Water (2:10, v/v ratio of water:homogenate) was mixed with the homogenate and allowed to stand for 10 min for full phase separation to occur. The organic layer (bottom phase) was collected in a round bottom flask. Residual lipids in the aqueous layer were subsequently extracted with 2:1 v/v chloroform/methanol (1:1 v/v aqueous to organic solvents) and allowed to stand for 10 min until phase separation occurred. The organic layer from the second extraction was combined with the previous collection in the same round bottom flask. The solvent of the combined organic layers was then evaporated to dryness in a rotary evaporator (initially 400 mbar, 100 rpm, 45°C, and pressure was continuously decreased to 0 mbar to remove any traces of remaining solvent). The lipid content was determined by weighing the difference of the empty round bottom flask at the start of the experiment and the same flask that contained the extracted lipids.

#### Chromatographic conditions for TAG analysis

The extracted lipids were then dissolved in chloroform to a final concentration of 2 mg/ml and analyzed using GC-FID. The analyses were performed on a PerkinElmer Clarus® 680 gas chromatograph (Beaconsfield, UK), equipped with a capillary ZB-1HT (100% dimethylpolysiloxane) column with a length of 12.5 m, an internal diameter of 0.32 mm, and a siloxane film thickness of 0.10 μm (Phenomenex, Inc, CA). The lipid samples (1 μl) were injected into a programmed temperature vaporization (PTV) split injector (split ratio of 1:50). The following PTV temperature program for the injector port was adopted: 60°C, held for 0.2 min, increased to 370°C at the maximum heating rate (∼125°C/min), and this temperature was held for 5.0 min. The following were the oven conditions: 250°C, held for 2.0 min, heated up to 360°C at a rate of 3.8°C/min, and temperature held for 4.0 min. The carrier gas was helium (1.54 ml/min, constant flow), and the FID was kept at 370°C. The average concentration of each TAG was automatically determined by the instrument software, and statistical analyses were conducted using a one-way ANOVA to determine the significance (*P* < 0.05) of any differences in the TAG content between both nonpasteurized and pasteurized human milk.

### Particle size measurement

Particle size distributions of the fat globules from the redispersed nonpasteurized and pasteurized human milk samples were measured by laser light scattering using a Mastersizer 2000 (Malvern Panalytical, UK), equipped with a He-Ne red laser of wavelength 633 nm and a light-emitting diode blue laser of wavelength 466 nm. Light scattering from both lasers was combined to detect volume size distributions ranging from 10^−2^ to 10^4^ μm. Background measurements were taken with water flowing through the cell, and the milk samples were then added dropwise until the obscuration of the red laser was 5–10% (equivalent to the obscuration of the blue laser being 7–14%). The refractive indexes of the milk fat globules and water were taken to be 1.46 and 1.30, respectively. The absorbance of the milk fat globules was taken as 0.001 for analysis. The volume-weighted mean diameter of the particles was then recorded as D_4,3_ generated by the in-built instrument software based on calculated size distributions by volume.

### In vitro lipolysis

In vitro lipolysis was performed on nonpasteurized and pasteurized human milk using methods described previously (24, 25). The human milk samples were stored at −20°C and thawed immediately prior to digestion. The human milk samples were redispersed and added into a thermostated glass vessel (maintained at a constant temperature of 37°C) connected to a pH stat autotitrator (Metrohm® AG) under constant magnetic stirring. The apparatus was connected to a computer and operated using Tiamo 2.0 software (Metrohm®). To simulate the gastric environment, the pH of the sample was adjusted to 3.000 ± 0.003 using 5.0 M HCl. Pepsin (13.9 mg) and fungal gastric lipase (27.8 mg) were then added to the sample. After an hour of gastric digestion at 37°C, the pH of the sample was then adjusted using HCl or NaOH (0.2–5.0 M) to a value of 6.500 ± 0.003 to mimic intestinal conditions before 2.25 ml of reconstituted pancreatic lipase suspension was injected into the sample. The lipase suspension was prepared by dispersing pancreatin in water followed by centrifugation before freeze-drying the supernatant to provide a powder form. This freeze-dried pancreatic lipase was dispersed in the digestion buffer (50 mM Trizma-maleate buffer at pH 6.5, which also contained 5 mM CaCl_2_.2H_2_O, 150 mM sodium chloride, and 6 mM sodium azide). The activity of pancreatic lipase approximates 700 tributyrin unit/ml of digest (measured independently by adding 2 ml of reconstituted lipase solution to 6 g of tributyrin stirred vigorously with 18 ml of digestion buffer).

Throughout the digestion of human milk lipids, 0.2 M NaOH was added to maintain the pH at 6.5 to counter the decrease in pH as a result of the liberation of FFAs. Assuming that the consumption of NaOH was only through the evolution of ionized FFAs by lipolysis, the amount of titrated (ionized) FFAs was determined following the subtraction of the volume of NaOH (required to maintain the pH at 6.5) from the blank digestion (human milk with the lipids removed). To prepare this blank digestion, 30 ml of human milk was centrifuged at 4,500 *g* at 4°C for 15 min before removing the lipid layer and collecting the supernatant (which was measured to contain 0.2% lipid using gravimetric analysis as described in the “Extraction of lipids” section). After 120 min of intestinal digestion of either human milk or the blank, the pH of the digested milk was increased to pH 9.0 using NaOH (“back titration”), for which the molar amount of NaOH required corresponds to the amount of unionized FFAs present at the end of digestion. Together with the amount of ionized FFAs determined earlier, the total amount of FFAs released during digestion was calculated. Based on the previous literature reports, the theoretical amounts of FFAs released by the nonpasteurized and pasteurized human milk samples were estimated to be around 3.72 and 2.87 mmol, respectively (26). Subsequently, the extent of digestion was calculated using Equation 1 assuming that 1 mol of TAG generates 2 mol of FFAs:

(1) $$\text{Extent of digestion }\left(\text{\%}\right)=\frac{\text{Ionized FFAs }\left(\text{mmol}\right)+\text{ unionized FFAs }\left(\text{mmol}\right)}{\text{Theoretical FFAs in nonpasteurized or pasteurized human milk}}\text{×}100\text{\%}$$

#### Effects of different concentrations of calcium and human milk fat content on digestion

The initial concentration of calcium in the digestion buffer was set at either 5 or 100 mM. For the digestion experiments using “high fat milk,” 20 ml of human milk and freeze-dried digestion buffers were added to the vessel. These freeze-dried digestion buffers were prepared by aliquoting 5 ml of either 5 or 100 mM calcium digestion buffer into 20 ml glass scintillation vials and freeze-dried for approximately 48 h using a VirTis Wizard 2.0 freeze dryer. The freeze-dried digestion buffers (285 mg) were weighed in 20 ml glass scintillation vials using an analytical balance before adding to the “high fat milk” samples. Based on the gravimetric analysis (see the “Extraction of lipids” section), nonpasteurized milk contained 5.9 g fat/100 ml of milk, whereas pasteurized milk contained 5.5 g fat/100 ml of milk. Thus, high fat nonpasteurized milk contained 5.9 w/v% fat and pasteurized milk contained 5.5 w/v% fat. For the experiments involving “low fat milk,” the human milk samples were diluted by 4-fold, that is, 5 ml of human milk was added to 15 ml of digestion buffer with added-calcium such that the final calcium concentrations were 5 or 100 mM. The fat content of nonpasteurized and pasteurized milk after dilution was approximately 1.5% and 1.4%, respectively. The digestion experiments were then conducted following the aforementioned protocol in the “In vitro lipolysis” section.

#### Preparation of bile salt micelles

1,2-Dioleoyl-*sn*-glycero-3-phosphocholine (102 mg) was weighed in a round bottom flask using an analytical balance before dissolving it in chloroform. The chloroform was then removed using a rotary evaporator (initially 400 mbar, 50 rpm, 40°C, and the pressure was continuously decreased to 0 mbar). Following this, 327 mg of sodium taurodeoxycholate hydrate was added to the dried extract. Approximately 90 ml of 50 mM Trizma-maleate buffer at pH 6.5 was then added to the same round bottom flask. The mixture was then bath sonicated and made up to a total volume of 100 ml. The final concentration of bile salt and phospholipid was 4.7 and 1.0 mM, respectively.

### SAXS: flow-through measurements

SAXS experiments were carried out on the SAXS/WAXS beamline at the Australian Synchrotron (ANSTO, Clayton, Victoria, Australia) (27) to determine real-time lipid liquid crystalline structure formation in human milk. The in vitro digestion apparatus described previously was coupled to the SAXS/WAXS beamline as explained previously (24). A peristaltic pump was used to aspirate a fraction of the digest at a flow rate of approximately 10 ml/min via silicone tubing through a 1.5 mm diameter quartz capillary mounted in the X-ray beam, and pancreatic lipase was added remotely using a syringe driver. An X-ray beam with a photon energy of 13.0 keV (wavelength, λ = 0.954 Å) was utilized in this investigation. A sample-to-detector distance of around 1.7 m (*q* range of approximately 0.01 < *q* < 1.09 Å^−1^) was used to monitor the liquid crystalline structures formed during digestion. *q* refers to the magnitude of the scattering vector or momentum transfer and expressed as in Equation 2:

(2) $$q=\left(\frac{4\pi }{\lambda }\right)\sin\left(\frac{2\theta }{2}\right)$$

where *λ* is the wavelength and 2*θ* is the scattering angle. The 2D SAXS patterns were recorded using a Pilatus 2M detector (active area 254 × 289 mm^2^, pixel size 172 × 172 μm^2^), with a 5 s acquisition time and 15 s delay between each measurement. Depending on the scattering profile, the phases of the liquid crystalline structures were identified based on their characteristic diffraction peaks as a function of time. 2D diffraction patterns recorded were converted into plots of scattered X-ray intensity against scattering vector (*q*) using the in-house developed software *Scatterbrain*. In these profiles, the presence of lamellar, hexagonal, and cubic liquid crystalline phases was determined based on their characteristic diffraction peaks (28). Subsequently, the lattice parameter or physical dimension of unit cells in the crystal lattice, *a*, was calculated using the equations based on the characteristic peak multiplier (*x*) and *q*-value of the corresponding peak (*q*_*peak*_):

$${\text{Lamellar and disordered inverse micellar L}}_{2}\text{ phase}:a=\frac{2\pi x}{q_{peak}}$$

where *x* = 1, 2, or 3

$${\text{Micellar cubic I}}_{2}\;\text{phase}:a=\frac{2\pi \surd x}{q_{peak}}$$

where *x* = 3, 8, or 11

$$\text{Hexagonal phase}:a=\frac{4\pi \surd x}{q_{peak}\surd 3}$$

where *x* = 1, 3, or 4.

### SAXS: precipitated calcium soaps

Calcium soaps of oleic acid and oleic acid mixed with monopalmitin (FFA:MAG ratio = 2:1) were prepared using a previously reported procedure (29). Briefly, the lipids were dissolved in absolute ethanol saturated with calcium chloride ([lipids] ∼20 mg/ml). Calcium soaps were then precipitated by adding ethanolic sodium hydroxide solution (0.117 M) such that the moles of sodium hydroxide matched the number of moles of lipid in the mixtures. The dispersions were loaded into special glass capillaries (Charles Supper, Natick, MA) and placed directly in the path of the X-ray beam (wavelength = 0.954 Å, photon energy = 13.0 keV) for measurement of their scattering patterns. 2D SAXS patterns were recorded with a Pilatus 1 M detector and an acquisition time of 1 s. The sample-detector distance was ∼1.6 m, which gave an accessible *q* range of 0.013–0.652 Å^−1^. An example of an X-ray scattering profile of calcium soaps is presented in supplemental Fig. S6.

### FFA composition analysis using GC-FID

#### Preparation of FAME from human milk

A solid phase extraction method as previously described by Agren *et al.* (30) was adopted to separate FFAs from the other components of human milk using a single aminopropyl column. Briefly, 500 mg/6 ml ISOLUTE® aminopropyl columns (Biotage, Uppsala, Sweden) were washed with acetone/water (7:1, v/v) and hexane. The digested human milk aliquots (1 ml each collected during digestion described in the “In vitro lipolysis” section with no back-titration step and lipolysis inhibited with 1 v/v% 0.5 M 4-bromophenyl boronic acid in methanol) were dissolved in 4.84 ml hexane/0.145 ml tert-butyl methyl ether/0.0145 ml acetic acid (100:3:0.3, v/v/v) mixture and applied to the column after centrifugation. The FFAs were eluted with chloroform/methanol/acetic acid (100:2:2, v/v/v) and collected in round bottom flasks. The collected FFAs were then evaporated to dryness in a rotary evaporator (initially 400 mbar, 50 rpm, 40°C, and the pressure was continuously decreased to 0 mbar to remove any traces of volatile solvent remaining). Following this, 200 μl of hexane was added to the dried extract.

To derivatize the FFAs into FAME, methanolic HCl (3 M) was used as the derivatizing agent. In an amber glass vial equipped with rubber-lined screw caps, the FFAs dissolved in hexane were added to 240 μl of internal standard FAME C11:0, prepared in the same solvent, 240 μl internal standard TAG C13:0, 1.6 ml of methanol, 1.6 ml of methanolic HCl, and 667 μl of hexane. The vials were firmly capped, shaken vigorously, and heated at 100°C for 1 h. After cooling down to room temperature, 1.56 ml of water was added and shaken vigorously before centrifugation at 1,200 *g* for 5 min. The upper organic layer was then transferred into GC vials for analysis.

#### Chromatographic conditions for FAME analysis

A polar column (70% cyanopropyl polysilphenylene-siloxane) with a length of 30 m, an internal diameter of 0.25 mm, and a film thickness of 0.25 μm was used for the analyses of free FAMEs (Trajan Scientific Pty Ltd, Australia). The samples (1 μl) were injected into a PTV split injector (split ratio of 1:50). The temperature of the injector port was maintained at 250°C throughout the run. The following were the oven conditions: 60°C, held for 2.0 min, increased to 200°C at 10°C/min, increased to 240°C at 5°C/min, and temperature held for 1.0 min. The carrier gas was helium (1.00 ml/min, constant flow), and the FID was kept at 250°C. The average concentration of each FAME was automatically determined by the instrument software. Following this, the average concentration of each FFA was calculated using the response factor as described in the electronic supplemental data and supplemental Table S1. The variation in the amount of each FFA released during the digestion of nonpasteurized and pasteurized human milk was subsequently determined using principal component (PC) analysis (PCA) in Orange software (version 3.22.0) developed by the University of Ljubljana (31).

## Results

### Effects of calcium, fat content, and bile salt micelles on the digestibility of human milk

**Fig. 2:**
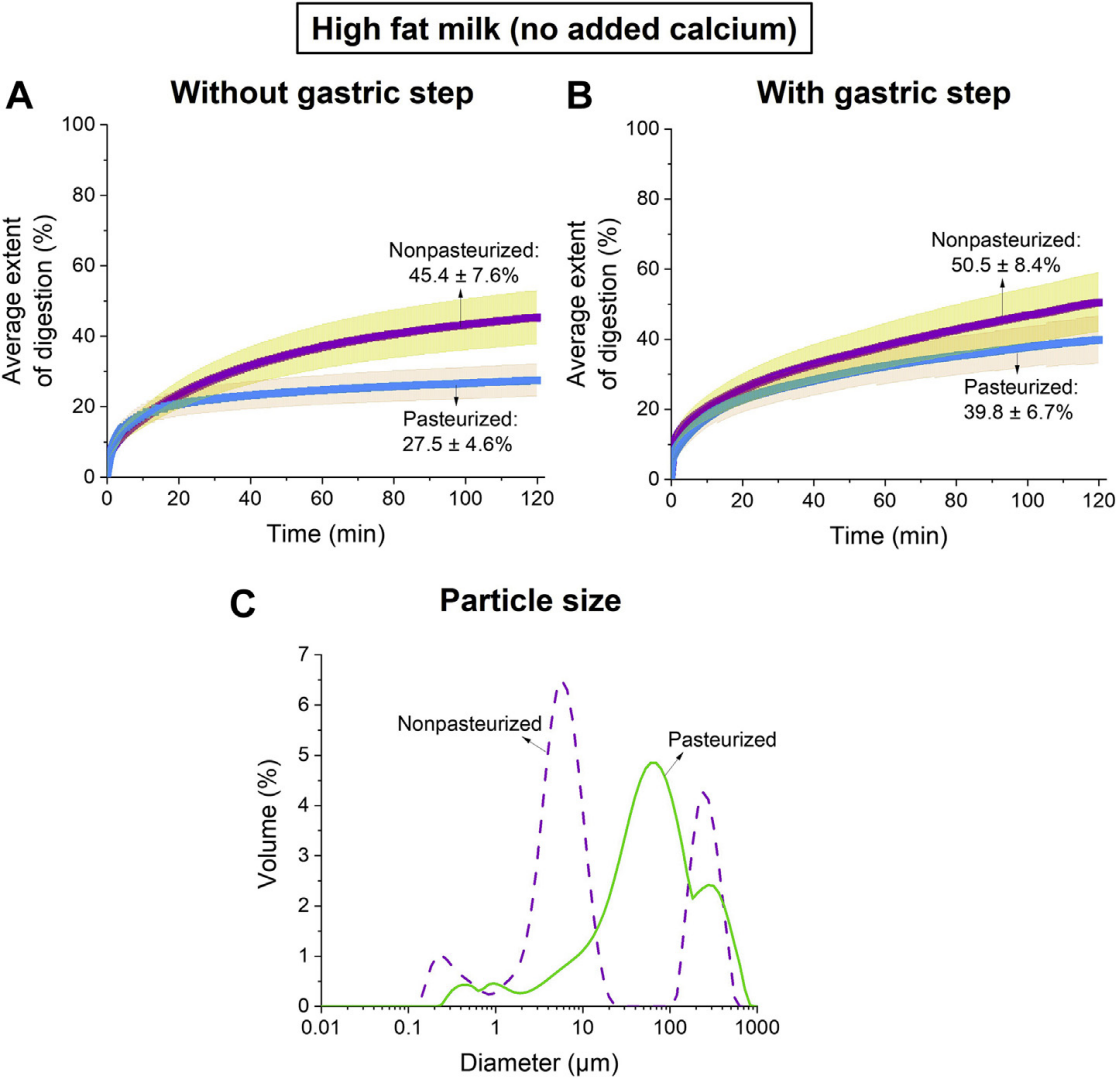
Extent of digestion of high fat nonpasteurized human milk (purple with yellow error bars; 5.9% fat) and high fat pasteurized human milk (blue with orange error bars; 5.5% fat) by pancreatic lipase at pH 6.5 (A) without a gastric predigestion step and (B) with a gastric digestion step. C: Particle size distributions of nonpasteurized (purple dashed line) and pasteurized human milk (green solid line).

The formation of self-assembled structures during digestion is dependent on the extent of conversion of the nonpolar triglycerides to polar monoglycerides and FFAs. Thus, it is important that digestion is as close to complete as possible for the findings to represent the potential in vivo situation. Initially, (as-supplied) nonpasteurized human milk with a fat content of 5.9% and pasteurized human milk containing 5.5% fat were digested without digestion buffer, that is, no added calcium and salts. The extents of digestion were low; only 45.4 ± 7.6% and 27.5 ± 4.6% of the lipids in nonpasteurized milk and pasteurized milk were digested, respectively, in the simulated small intestinal condition at pH 6.5 (Fig. 2A). However, when a gastric digestion step (pH 3.0) was introduced prior to the intestinal digestion (pH 6.5), the extents of digestion of nonpasteurized and pasteurized milk increased to 50.5 ± 8.4% and 39.8 ± 6.7%, respectively (Fig. 2B). Particle size distributions of the nonpasteurized and pasteurized human milk are shown in Fig. 2C, with an averaged D_4,3_ of 53.2 and 118 μm, respectively.

**Fig. 3:**
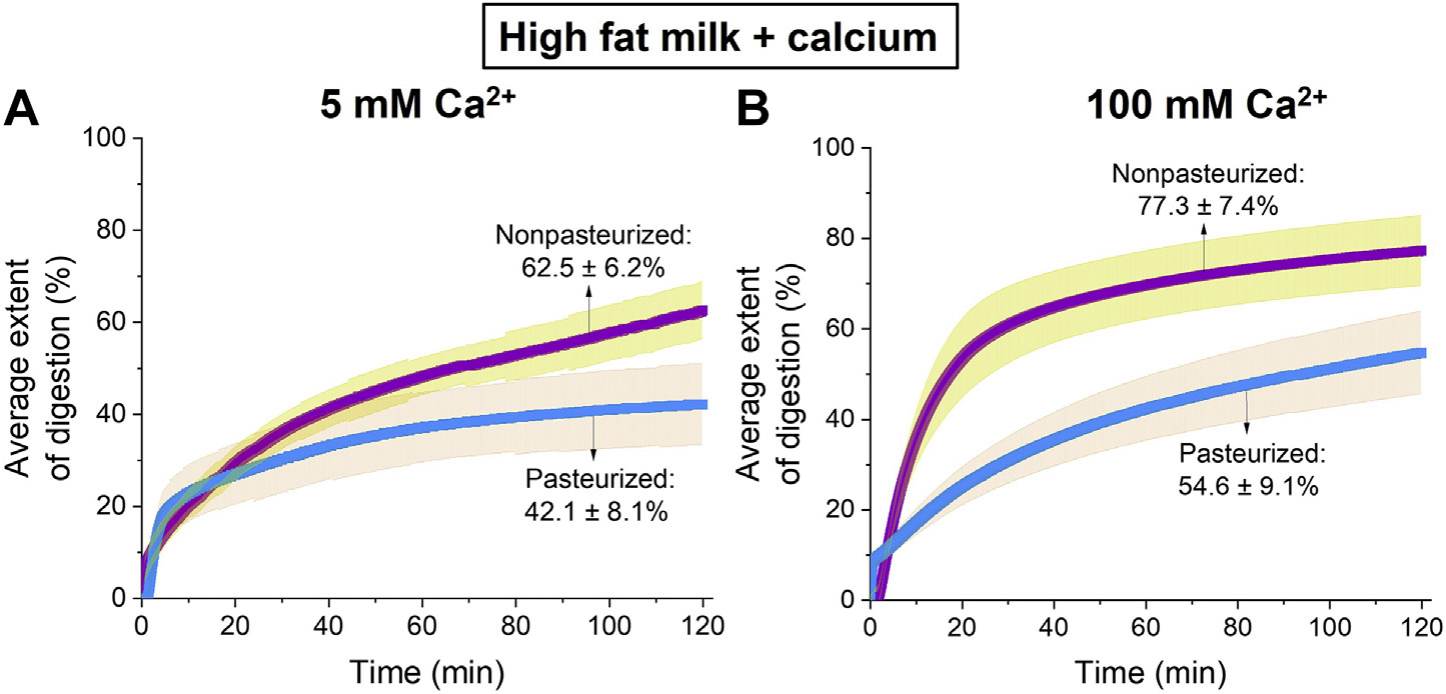
Extent of digestion of high fat nonpasteurized human milk (purple with yellow error bars; 5.9% fat) and high fat pasteurized human milk (blue with orange error bars; 5.5% fat) when (A) 5 mM and (B) 100 mM calcium were included in the digestion buffers.

To drive the extent of digestion further towards completion, calcium was added during the digestion of human milk at a final concentration of 5 and 100 mM. The results in Fig. 3A, B show that the digestibility of human milk, both pasteurized and nonpasteurized, was affected by the calcium concentration. With an added calcium concentration of 5 mM, 62.5 ± 6.2% and 42.1 ± 8.1% of the nonpasteurized and pasteurized human milk was digested, respectively (Fig. 3A). When the added concentration of calcium was increased from 5 to 100 mM, the final extent of digestion of nonpasteurized milk increased to 77.3 ± 7.4% and that of pasteurized milk to 54.6 ± 9.1% (Fig. 3B).

**Fig. 4:**
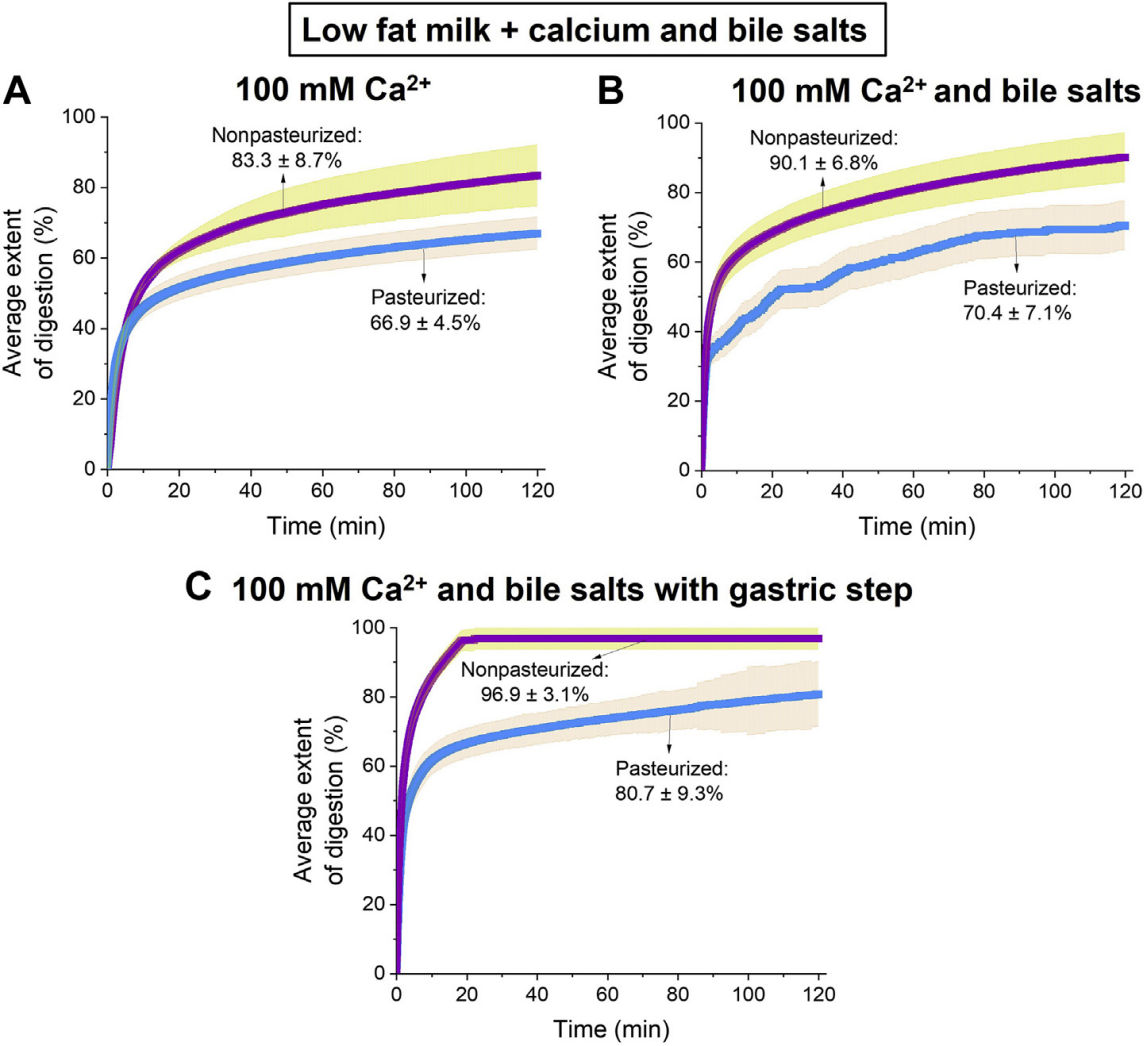
Extent of digestion of low fat nonpasteurized human milk (purple with yellow error bars; 1.5% fat) and low fat pasteurized human milk (blue with orange error bars; 1.4% fat) at pH 6.5 when (A) 100 mM calcium was added into the digestion buffers. The extents of digestion of these human milk samples were subsequently determined after adding bile salt micelles (B) without gastric step and (C) with gastric step.

Since incomplete digestion was still observed, the samples were diluted 4-fold to reduce the fat content of nonpasteurized and pasteurized human milk relative to the calcium. While the dilution increased the extent of digestion (Fig. 4A), the addition of bile salt micelles to the mixtures led to a significant increase in the extent of digestion of both types of human milk (to 90.1 ± 6.8% for nonpasteurized human milk and 70.4 ± 7.1% for pasteurized human milk) (Fig. 4B). Moreover, when the gastric step was included in the digestion, the nonpasteurized milk attained essentially complete digestion of 96.9 ± 3.1% within 20 min of digestion, whereas that of pasteurized milk was 80.7 ± 9.3% after 120 min of digestion (Fig. 4C).

### Correlation between extent of digestion and self-assembly of lipids

**Fig. 5:**
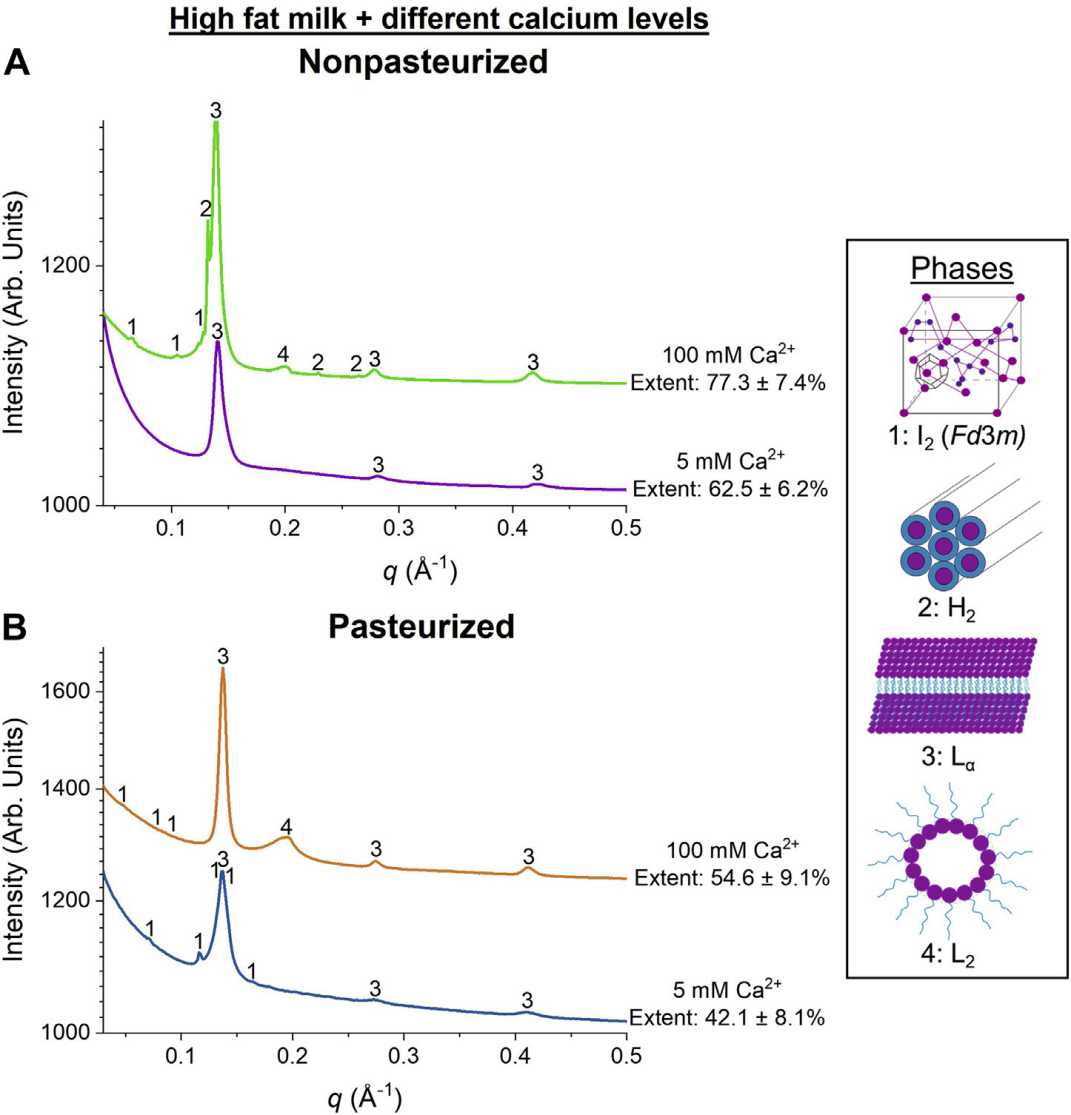
SAXS profiles for the digestion of (A) high fat (5.9% fat) nonpasteurized human milk with 5 mM calcium (purple line) and 100 mM calcium (green line) and (B) high fat (5.5% fat) pasteurized human milk with 5 mM calcium (blue line) and 100 mM calcium (orange line) after the addition of lipase at pH 6.5, 37°C. “1” represents the cubic micellar I_2_ phase (*Fd3m* space group), with peak *q* ratios of √3:√8:√11, “2” is annotated as the hexagonal H_2_ phase with peak *q* ratios of 1:√3:√4, and “3” and “4” represent the lamellar L_α_ phase with peak q ratios of 1: 2: 3 and disordered inverse micellar L_2_ phase, respectively.

**Fig. 6:**
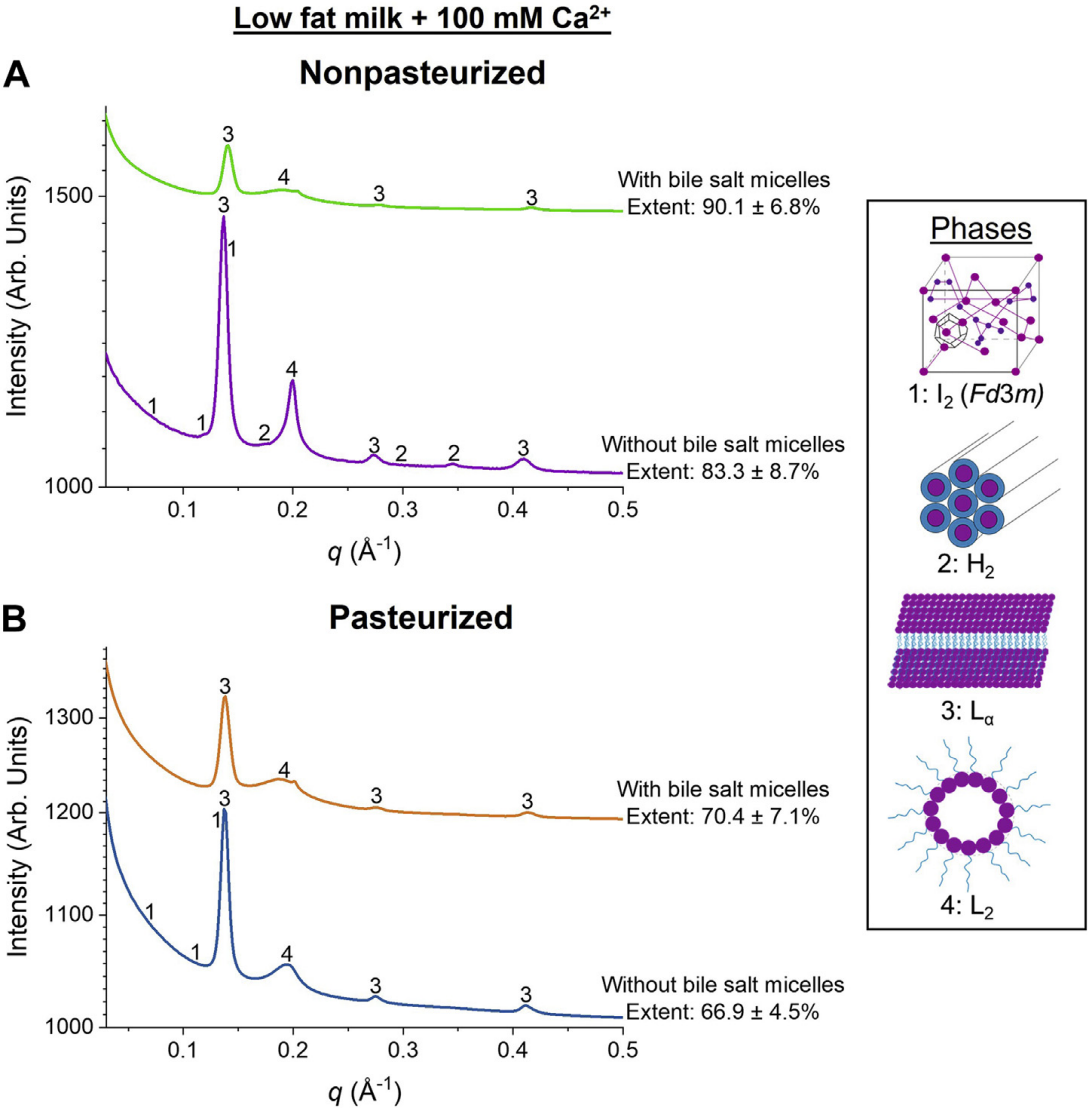
SAXS profiles for the digestion of (A) low fat (1.5% fat) nonpasteurized human milk with 100 mM calcium in the absence of bile salt micelles (purple line) and with bile salt micelles (green line). B: SAXS profiles for the digestion of low fat (1.4% fat) pasteurized human milk with 100 mM calcium in the absence of bile salt micelles (blue line) and with bile salt micelles (orange line) after the addition of lipase at pH 6.5, 37°C. “1” represents the cubic micellar I_2_ phase (*Fd3m* space group), with peak *q* ratios of √3:√8:√11, “2” is annotated as the hexagonal H_2_ phase with peak *q* ratios of 1:√3:√4, and “3” and “4” represent the lamellar L_α_ phase with peak q ratios of 1: 2: 3 and disordered inverse micellar L_2_ phase, respectively.

It has been shown previously that a series of lipid liquid crystalline structures appear during the digestion of human milk as a result of the self-assembly of amphiphilic diacylglycerols, MAGs, and FFAs (22, 32). These lipid liquid crystalline phases were determined based on the positional distribution of their respective diffraction peaks using the equations provided in the Materials and methods section. The SAXS profiles were plotted as a function of extent of digestion (Fig. 5, Fig. 6).

Following the addition of pancreatic lipase to nonpasteurized human milk with 5 mM calcium, diffraction peaks corresponding to a lamellar structure with a lattice parameter of 46–47 Å were observed and remained persistent throughout digestion (Fig. 5A). This phase is associated with the formation of calcium soaps through the binding of calcium ions that are naturally found in milk and the digestion buffer used in these in vitro experiments (29). To mimic the upper intestinal environment, the pH of the digestion buffer was set to 6.5, which resulted in the partial deprotonation of FFAs generated by lipolysis that are reported to have pKa values of 4–9 (33). However, as the concentration of calcium was increased from 5 to 100 mM in the digestion buffers, not only was a lamellar phase also present during digestion but also higher order lipid liquid crystalline phases, such as a cubic micellar I_2_ phase (*Fd*3*m* space group) (*q* = 0.065, 0.106, and 0.124 Å^−1^) and a hexagonal H_2_ phase (*q* = 0.134, 0.232, and 0.268 Å^−1^) were observed (Fig. 5A). Moreover, a diffraction peak (*q* = 0.198 Å^−1^) that is typically associated with a disordered inverse micellar L_2_ phase was also observed when 100 mM calcium was included in the digestion buffer (Fig. 5A).

In contrast, the digestion of pasteurized human milk at 5 mM calcium resulted in the formation of a cubic micellar I_2_ phase (*Fd*3*m* space group) with peaks at *q* = 0.0712, 0.116, 0.136, 0.142, and 0.164 Å^−1^ in addition to a persistent lamellar phase (Fig. 5B). However, when the concentration of calcium was increased to 100 mM, no additional phases were observed for pasteurized human milk (Fig. 5B) unlike nonpasteurized human milk (Fig. 5A). Instead, a peak at approximately *q* = 0.189 Å^−1^ that is indicative of an L_2_ phase was again observed during the digestion of pasteurized human milk at the same concentration of calcium (Fig. 5B).

The self-assembly of lipids during the digestion of nonpasteurized and pasteurized human milk was the same when the samples were diluted to generate “low fat milk” (Fig. 6A, B). However, when bile salt micelles were introduced to the systems, the nonlamellar phases were not observed during digestion (Fig. 6A, B). Instead, a weak broad peak indicative of a disordered inverse micellar L_2_ phase with the peak centred around *q* = 0.187–0.205 Å was observed for both types of human milk.

A summary of the extents of lipid digestion and the corresponding types of lipid liquid crystalline structures formed during the digestion of nonpasteurized and pasteurized human milk are presented in Table 1.

**Table 1:** Summary table of the different digestion conditions of nonpasteurized and pasteurized human milk and the corresponding particle size, maximum extent of digestion, and the liquid crystal structures formed

Digestion Conditions	D_4,3_ (μm)	Maximum Extent of Digestion (%)	Phases and Lattice Parameter (Å)
Nonpasteurized human milk			
High fat (no added calcium)	75.5	45.4 ± 7.6	L_α_: 45
High fat + 5 mM Ca^2+^	26.9	62.5 ± 6.2	L_α_: 46
High fat + 100 mM Ca^2+^	24.0	77.3 ± 7.4	L_α_: 47 *Fd*3*m*: 167 H_2_: 56 L_2_: 37
Low fat + 100 mM Ca^2+^	17.2	83.3 ± 8.7	L_α_: 46 *Fd*3*m*: 164 H_2_: 55 L_2_: 40
Low fat + 100 mM Ca^2+^ with bile salt micelles	14.0	90.1 ± 6.8	L_α_: 45 L_2_: 38
Pasteurized human milk			
High fat (no added calcium)	118.9	27.5 ± 4.6	L_α_: 46
High fat + 5 mM Ca^2+^	72.5	42.1 ± 8.1	L_α_: 46 *Fd*3*m*: 164
High fat + 100 mM Ca^2+^	60.3	54.6 ± 9.1	L_α_: 47 *Fd*3*m*: 166 L_2_: 38
Low fat + 100 mM Ca^2+^	43.2	66.9 ± 4.5	L_α_: 46 *Fd*3*m*: 167 L_2_: 40
Low fat + 100 mM Ca^2+^ with bile salt micelles	29.1	70.4 ± 7.1	L_α_: 47 L_2_: 39

### FFA composition during the digestion of human milk

Human milk lipid extracts were analyzed using GC-FID. The chromatographic profiles and relative amounts of TAGs in nonpasteurized and pasteurized human milk are provided in the supplemental data (supplemental Figs. S1 and S2 and supplemental Table S2). Although the extent of digestion of nonpasteurized and pasteurized human milk was the greatest when 100 mM calcium and bile salts were added, the self-assembly of lipids during digestion was similar for both types of milk (Fig. 6). Instead, to determine whether the FFA composition does have an influence on the self-assembly of lipids during digestion, the relative percentages of FFAs released from nonpasteurized and pasteurized human milk were determined when 100 mM calcium was added to the systems, without bile salts.

**Fig. 7:**
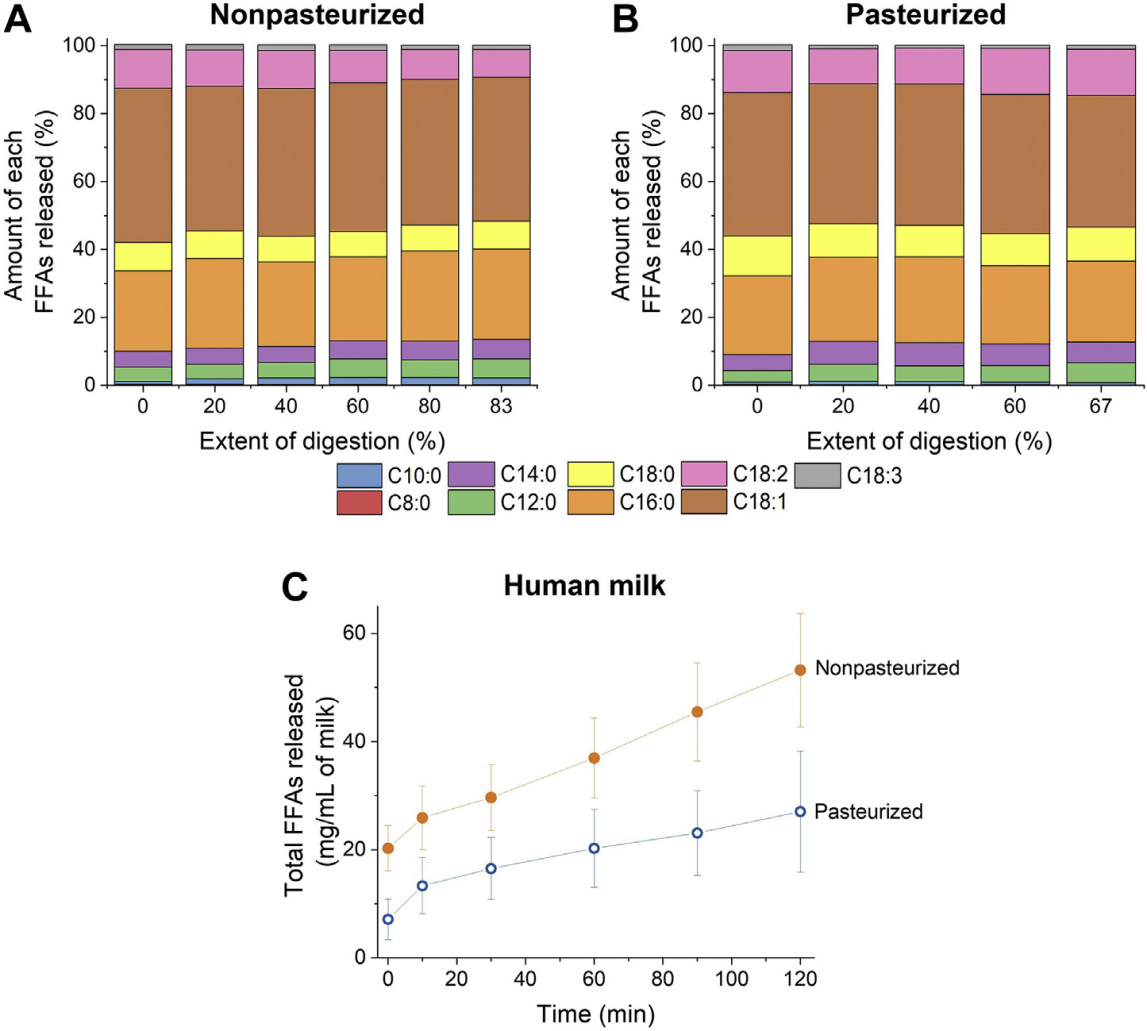
Amount of each FFA released plotted as a function of extent of digestion for (A) low fat (1.5% fat) nonpasteurized and (B) low fat (1.4% fat) pasteurized human milk with 100 mM calcium. C: Total amount of FFAs released from nonpasteurized (filled orange circles) and pasteurized (hollow blue circles) human milk. Results are n = 3 ± SD of three separate injections, and human milk samples were pooled from a single donor.

In both nonpasteurized and pasteurized human milk, oleic acid (C18:1) made up the largest fraction of FFAs released at each stage of digestion (Fig. 7A, B). The percentages of long-chain polyunsaturated FFAs, including linoleic acid (C18:2) and stearic acid (C18:0), increased as the digestion progressed. However, palmitic acid (C16:0) still accounted for a large proportion of FFAs released being the second largest fraction after C18:1 (Fig. 7A, B). Looking at the total amount of FFAs released at each time point, it can be seen that nonpasteurized human milk released a greater amount of FFAs than pasteurized human milk (Fig. 7C).

### Relationship between FFA composition and self-assembly during digestion

**Fig. 8:**
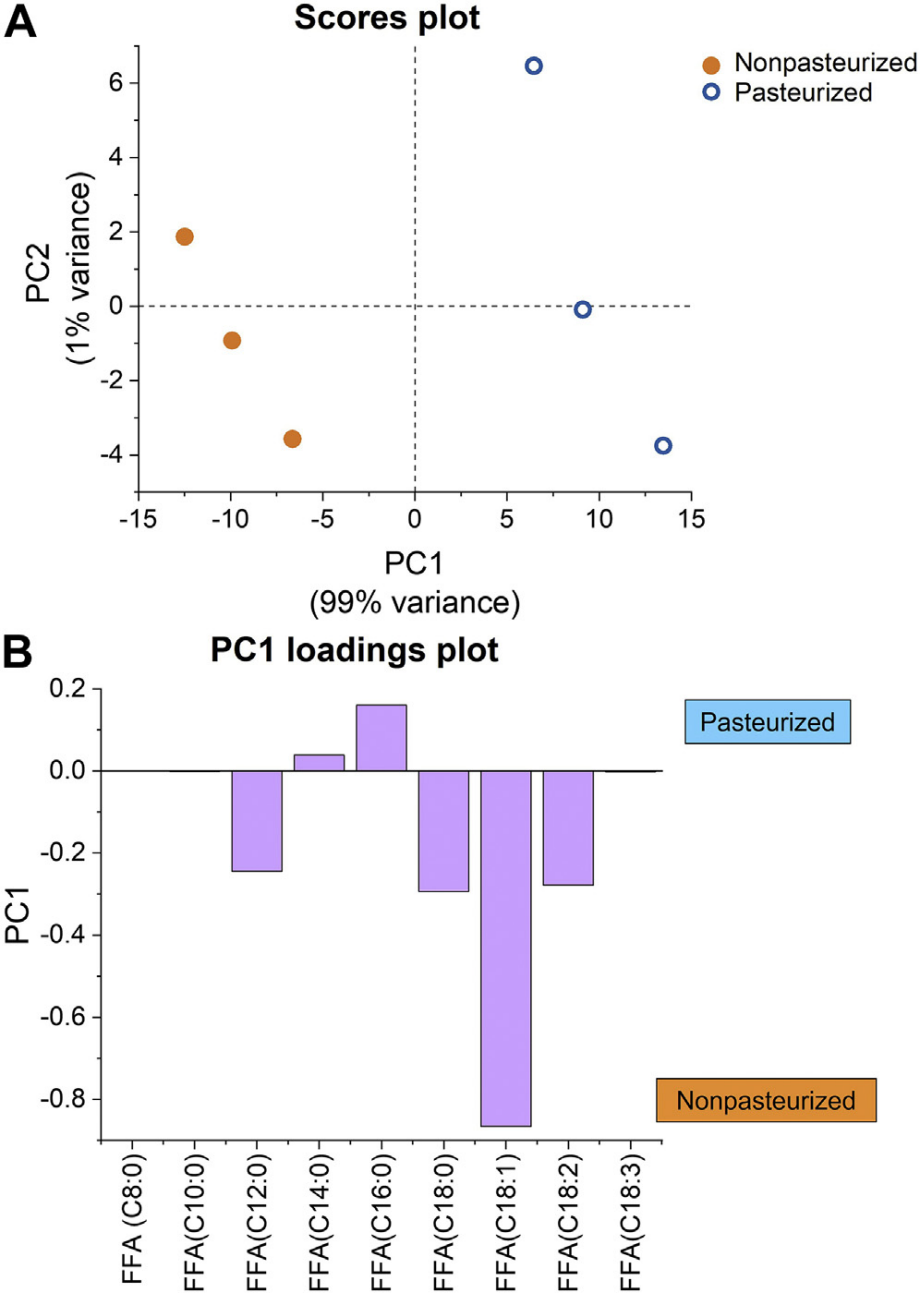
A: PCA scores plot separating nonpasteurized from pasteurized human milk based on the respective percentages of FFAs released during digestion after 120 min. B: PC1 loading plot compares the percentages of FFAs based on the scores plot. Experiments were conducted in triplicate (samples pooled from a single donor), leading to three individual data points in the scores plot.

PCA was used to probe the relationship between the amount of each FFA released during digestion from nonpasteurized and pasteurized human milk (Fig. 8). Supplemental Table S3 summarizes the average concentration and weight percent of each FFA released after 120 min of digestion of nonpasteurized and pasteurized human milk. The percentages of FFAs released after 120 min of digestion for both types of human milk were used to graph a scores plot (PC1 vs. PC2) (Fig. 8A). Based on this plot alone, it can be seen that there is a separation between both types of human milk, with nonpasteurized milk displaying PC1 values <1 and pasteurized milk showing PC1 values >1. The loading plot for PC1, which separates both types of human milk, shows that lauric acid (C12:0), stearic acid (C18:0), oleic acid (C18:1), and linoleic acid (C18:2) are in greater abundance in nonpasteurized than pasteurized human milk (Fig. 8B), which was also evident from supplemental Table S3. Since the PC1 loading plot accounts for 99% of the variance, other differences in the FFA content of the two milks (accounted for by PC2) were minor (supplemental Fig. S3).

## Discussion

Human milk lipids are considered to be one of the most complex dietary fats with beneficial nutritional properties for infants (34). Despite variations in the lipid and FA compositions because of disparities in diet, age, and parity, human milk is considered as a colloidal nutrient delivery system whose function depends on the digestion of lipids in the infant's GIT. TAGs comprise approximately 98 wt% of the total lipid fraction, which provides 45–55% of the total energy intake in infants. Regardless of the factors that might influence the composition of lipids and FAs in human milk, the digestion of lipids and subsequently absorption of FFAs is important in the development of the brain and central nervous system in infants (19, 35). Moreover, these digested lipids are able to self-assemble into colloidal structures (10) and potentially act as chaperones to deliver lipophilic nutrients to the systemic circulation.

Since the fate of lipophilic nutrients in the GIT is essential for the development of organs in infants, evaluating the factors that are able to drive the digestion process toward completion is important. First, a gastric step was introduced prior to the intestinal step with an aim to increase the extent of digestion. In adults, intestinal digestion contributes the majority of the lipolysis of triglyceride to products; however, in the infant, gastric digestion is more important. In the current study, nonpasteurized and pasteurized human milk were only partially digested after including a gastric step (Fig. 2A, B). Considering the limited contribution of gastric digestion, it was noted that several in vitro digestion studies have demonstrated the use of calcium as a tool to drive the digestion process of long-chain TAGs (36, 37, 38). As the concentration of calcium ions in a mixture is increased, more calcium is available to interact with the FFAs that are located on the oil-water interface of the lipid droplets, thereby exposing fresh TAGs to be digested by pancreatic lipase (20). This was seen in the present study as both human milks displayed an increase in the extent of lipid digestion as the concentration of calcium in the digestion buffers was increased (Fig. 3A, B). In addition, as the human milk samples were diluted with the digestion buffer, the fat content was lowered, thereby increasing the ratio of calcium:fat. Thus, this further amplified the increase in extent of digestion of nonpasteurized and pasteurized human milk (Fig. 4A).

Aside from increasing the concentration of calcium to enhance the extent of lipid digestion, adding bile salt micelles to the samples can stimulate the digestion of lipids toward completion (Fig. 4B, C). While lipases in the stomach and small intestine digest TAGs, bile salts aid in the emulsification of chime, thereby decreasing the particle size of the lipid droplets (supplemental Fig. S4). In turn, this increases the overall surface area for lipolysis and thus catalyzes the activity of lipases through the removal of digested products from the oil-water interface in mixed micelles (39). The concentration of bile salts has been reported to vary according to age with adults having a bile salt concentration of 5–25 mM and infants having only 1–5 mM bile salts intraduodenally (40). Based on several studies, the pooled data from 225 infants were calculated to be 4.7 ± 1.4 mM in the fasted state (41, 42, 43). Therefore, this bile salt concentration was used in these digestion experiments. However, despite adding bile salts to high fat pasteurized milk (5.5% fat) with 5 mM calcium, the sample was only partially digested (supplemental Fig. S5). Instead, both types of human milk attained almost complete digestion when a high calcium to milk fat ratio and bile salts were added to the systems (Fig. 4B, C).

It should be noted that in vitro digestion models are closed systems, characterized by an absence of a sink to remove the digestion products (25). As a result, this increases the concentration of lipid digestion products compared with what may be expected in vivo, and in turn, leads to the accumulation of digestion products at the oil-water interface (44, 45), which restricts further digestion from occurring. In contrast, in the in vivo situation, unlike the in vitro model, the presence of a sink enables the removal of digestion products through absorption by the epithelial cells in the small intestine. The concentration of calcium used in these experiments was either 5 or 100 mM, but the typical concentration of calcium in newborns and premature infants in the fasted state ranges from 1.55 to 2.75 mM with premature babies often experiencing hypocalcemia and thereby having a calcium concentration of <2.00 mM in their body fluids (46). Moreover, it was reported that the concentration of calcium in mature human milk was 6.25–7.40 mM (47, 48, 49). Hence, although the physiological concentrations of calcium are lower than those used in these in vitro digestion experiments, infants are still able to digest and absorb human milk lipids.

It has been shown previously that during the digestion of mammalian milks and infant formulae that the initial formation of calcium soaps typically precedes the formation of nonlamellar liquid crystalline phases, the identity of which depends both on the lipid composition and the extent of digestion (32, 50, 51). At 5 mM calcium, nonpasteurized human milk reached a greater extent of digestion than pasteurized human milk (Fig. 3A). This difference could be attributed to the lack of homogeneity of the pasteurized human milk samples, which had partially phase separated during the pasteurization process and were redispersed prior to digestion. The phase separation might have led to a larger particle size distribution of lipid droplets from pasteurized than nonpasteurized human milk (Fig. 2C).

As the concentration of calcium was increased to 100 mM, additional inverse micellar cubic I_2_ (*Fd*3*m* spacegroup) and hexagonal H_2_ phases were formed for nonpasteurized milk, but no changes were observed for pasteurized milk (Fig. 5A, B and Table 1). This could be due to differences in the extents of digestion for both milks. The maximum extent of digestion of nonpasteurized milk was 77.3% when the 100 mM of calcium was added to the digestion buffer (Fig. 3B). This led to the formation of nonlamellar structures (I_2_ and H_2_ phases) observed (Fig. 5A and Table 1). In contrast, the extent of digestion of pasteurized milk, which was 54.6%, was less than that of nonpasteurized human milk with the same added concentration of calcium (Fig. 3B). Consequently, no changes in the types of lipid liquid crystalline structures were observed (Fig. 5B and Table 1). The extent of digestion therefore appears to play a major role in the formation of additional self-assembled structures during the digestion of human milk.

Although no changes in structural behavior were observed as the fat content of nonpasteurized and pasteurized human milk was reduced (Fig. 6A, B), the presence of bile salts meant that nonlamellar phases were not observed during digestion (Fig. 6A, B). The difference in self-assembly of lipids indicates an interaction between the bile salts and internal particle structure of the digested lipids, with the oil phase becoming more hydrophilic (22). Instead, a weak broad peak characteristic of a disordered inverse micellar L_2_ phase appeared for both human milks. Although the addition of bile salts increased the extent of digestion for both types of samples (Fig. 4B), the facial amphiphilic moiety of bile salts enables the molecule to incorporate into lipid bilayers and mixed micelles during digestion. As this occurs, the critical packing parameter of the lipid liquid crystalline structures is reduced, thereby favoring a phase transition to a more lamellar phase (10, 52). This observtion is based on the absence of nonlamellar (I_2_ and hexagonal) phases, leaving only the formation of a lamellar phase and disordered L_2_ phase, which is indicated by the broad diffraction peak (Fig. 6). In addition, because of the high concentration of calcium in the digesting system, this caused the mixture of monopalmitin/oleic acid (1:2 mol/mol) to precipitate in a manner analogous to the same mixture in basic ethanol saturated with calcium chloride (supplemental Fig. S6). Consequently, the aggregation of free oleic acid in the presence of monopalmitin could have also contributed to the formation of this L_2_ phase.

Both types of human milk displayed an abundance of the C52 TAG species (supplemental Fig. S2). Within this C52 TAG, it can be inferred that the predominant FA composition is O/P/O (C18:1/C16:0/C18:1), which is consistent with previous reports (53, 54, 55). This unique O/P/O structure is vital to the nutrition and development of infants (54). While oleic acid displays preference for the *sn*-1 and *sn*-3 positions, palmitic acid is located primarily at the *sn*-2 position. During digestion, pancreatic lipase and BSSL hydrolyze the TAGs to produce 2-MAGs and FFAs. In the case of abundant O/P/O TAGs, the two outer oleic acids are first removed by the aforementioned enzymes, liberating two free oleic acids and leaving 2-monopalmitin (16). The selective esterification of the FFAs minimizes calcium loss and improves absorption of FFAs, contributing to important benefits, such as improved energy intake, healthy gut bacteria, and increased bone mineral density in infants (13, 14). Therefore, the highly selective distribution of FAs along the glycerol backbone of a TAG molecule thus underlines the biological significance of the structure of milk TAGs secreted by the mammary gland.

It has been previously illustrated that there is a direct correlation between lipid composition and the lipid liquid crystalline structures that form during digestion (32). This can also be seen in this study, whereby differences in structural behavior of lipids during digestion were observed because of differences in the amount and composition of FFAs released from nonpasteurized and pasteurized milk. Nonpasteurized human milk released a greater total amount of FFAs at the end of 120 min intestinal digestion as compared with pasteurized milk (Fig. 7C), with the PCA loading plot highlighting a difference between both milks (Fig. 8). While the total FA composition between both types of human milk before digestion is similar (supplemental Fig. S7), the greater release of medium-chain and long-chain FFAs (lauric acid [C12:0], stearic acid [C18:0], oleic acid [C18:1], and linoleic acid [C18:2]) from nonpasteurized than pasteurized milk (Fig. 8B) could have contributed to the differences in colloidal structures formed during digestion (Figs. 5 and 6). Pham *et al.* (32) have demonstrated that systems comprising medium-chain and long-chain FAs would result in the formation of nonlamellar structures such as the H_2_ and I_2_ phases.

The greater release of these FFAs from nonpasteurized than pasteurized milk could be due to the presence of active BSSL in the nonpasteurized human milk. During pasteurization, the heating step at 62.5°C suppresses the activity of BSSL (56, 57). As a result, the inactivation of BSSL in pasteurized milk could have led to a relatively smaller amount of the aforementioned FFAs being released than in the case of nonpasteurized milk. Since the intestinal bioaccessibility of these FFAs have nutritional and clinical impacts on infants, especially preterm infants, the lower amount of FFAs released from pasteurized than nonpasteurized milk could in turn lead to unfavorable consequences for the absorption of lipids by infants. For example, while medium-chain FAs are known to have an important role in maintaining the microbiota in infants, long-chain polyunsaturated FAs are involved in the development of the brain and stimulating rapid tissue growth (18). A study by Williamson *et al.* (47) showed that infants fed with pasteurized milk demonstrated impaired digestion and absorption of lipids (53.7% absorption), whereas the absorption of lipids was found to be 73.6% for infants fed with nonpasteurized human milk. This finding correlates with our study as nonpasteurized milk had a greater extent of digestion than pasteurized milk, thereby leading to greater amounts of FFAs being released. Hence, pasteurization of human milk could potentially limit the ability of these digested FFAs to exert their beneficial effects and thus where possible, nonpasteurized human milk is still the preferred feeding option for infants than pasteurized donor milk.

This article links the impact of pasteurization with particle size distribution and structural behavior, with the nonpasteurized and pasteurized human milk samples pooled from a single donor. As a result of obtaining these samples from a single donor, lipid compositions and percentages of FFAs were similar within the group of nonpasteurized milk. These results were also similar within the samples from pasteurized milk. However, these results were different when comparing the two sample groups of nonpasteurized and pasteurized milk as discussed earlier. Since it is known that the composition of human milk varies among mothers depending on diet, ethnicity, and other factors, future studies will establish the impact of differences in composition of different milk types on the self-assembly of lipids.

Previous studies have shown the ability of colloidal lipid structures to act as a reservoir for the dissolution of lipophilic drugs during digestion and deliver these drugs to the intestinal sites of absorption (58, 59, 60). The hexagonal and inverse micellar cubic I_2_ (*Fd*3*m* spacegroup) phases have been of great interest in the area of drug delivery as these phases have been shown to enhance the uptake of poorly water-soluble drugs in vivo. For instance, the formation of these colloidal structures during the digestion of milk lipids has been illustrated to improve the solubility of antimalarial drugs (OZ439 and ferroquine) and cinnarizine, which display poor solubility in undigested milk fat (58, 59, 60). Similarly, depending on the hydrophobicity/hydrophilicity of the bioactive molecules such as proteins and carbohydrates found in human milk, they would potentially be encapsulated either in the aqueous domain or within the lipid bilayers of the lipid liquid crystalline structures. In addition, the capacity for the colloids to deliver these nutrients can depend on their self-assembled structures. For example, although lamellar and the inverse micellar cubic phases can both encapsulate bioactive compounds, the capacity for these phases to deliver hydrophobic and hydrophilic compounds differ from each other. In contrast to a lamellar phase, inverse micellar cubic phases are more effective at encapsulating hydrophilic nutrients (such as digested proteins—peptides and amino acids) within the aqueous pores, hydrophobic components within the lipid matrix and amphiphilic nutrients at the oil-water interfaces (61, 62, 63, 64, 65).

Although both nonpasteurized and pasteurized milk formed an inverse micellar cubic I_2_ phase, only the digestion of nonpasteurized milk lipids resulted in the appearance of a hexagonal phase in the absence of bile salts (Figs. 5 and 6). The formation of the hexagonal phase during the digestion of nonpasteurized milk lipids could potentially accommodate additional bioactive molecules, which might not be possible for pasteurized milk. Furthermore, as heat treatments and homogenization processes during pasteurization might deactivate BSSL, this might impede the release of FFAs during digestion. Thus, the types of lipid liquid crystalline structures formed might be affected and potentially alter their capacity to accommodate bioactive molecules. The conditions used for any pasteurization method should therefore be taken into consideration to best preserve the bioactivity of enzymes and composition of lipids and FFAs released during digestion as these factors would dictate the structural behavior of milk lipids. Hence, the formation of these colloidal structures could be a pathway to understanding the ability of these structures to act as delivery vehicles to transport poorly water-soluble vitamins and nutrients to the systemic circulation, thereby potentially promoting the healthy development of infants.

## Data availability

All data are contained within the article.

## Supplemental data

This article contains supplemental data (66).

## Conflict of interest

The authors declare that they have no conflicts of interest with the contents of this article.

## References

[1] World Health Organization (2013).

[2] Oftedal O.T. (2012). The evolution of milk secretion and its ancient origins. Animal.

[3] Hylander M.A., Strobino D.M., Dhanireddy R. (1998). Human milk feedings and infection among very low birth weight infants. Pediatrics.

[4] Ouwehand A.C., Derrien M., de Vos W., Tiihonen K., Rautonen N. (2005). Prebiotics and other microbial substrates for gut functionality. Curr. Opin. Biotechnol..

[5] Lamberti L.M., Fischer, Walker C.L., Noiman A., Victora C., Black R.E. (2011). Breastfeeding and the risk for diarrhea morbidity and mortality. BMC Public Health.

[6] Nancy E.W. (2001). Donor human milk for preterm infants. J. Perinatol..

[7] Balmer S.E., Williams A.F. (1995). Guidelines for the establishment and operation of human milk banks in the UK. Arch. Dis. Child..

[8] Haschke F., Haiden N., Thakkar S.K. (2016). Nutritive and bioactive proteins in breastmilk. Ann. Nutr. Metab..

[9] Andersson Y., Sävman K., Bläckberg L., Hernell O. (2007). Pasteurization of mother's own milk reduces fat absorption and growth in preterm infants. Acta Pædiatr..

[10] Salentinig S., Sagalowicz L., Leser M.E., Tedeschi C., Glatter O. (2011). Transitions in the internal structure of lipid droplets during fat digestion. Soft Matter.

[11] Jensen R.G. (1995).

[12] Liu Z., Wang J., Cocks B.G., Rochfort S. (2017). Seasonal variation of triacylglycerol profile of bovine milk. Metabolites.

[13] Robles A., Jiménez M.J., Esteban L., González P.A., Martín L., Rodríguez A., Molina E. (2011). Enzymatic production of human milk fat substitutes containing palmitic and docosahexaenoic acids at sn-2 position and oleic acid at sn-1,3 positions. LWT - Food Sci. Technol..

[14] Filer L.J., Mattson F.H., Fomon S.J. (1969). Triglyceride configuration and fat absorption by the human infant. J. Nutr..

[15] Carey M.C., Small D.M., Bliss C.M. (1983). Lipid digestion and absorption. Annu. Rev. Physiol..

[16] John S.P., Martin C.C. (1979). Watching fat digestion. Science.

[17] Kalantzi L., Goumas K., Kalioras V., Abrahamsson B., Dressman J.B., Reppas C. (2006). Characterization of the human upper gastrointestinal contents under conditions simulating bioavailability/bioequivalence studies. Pharm. Res..

[18] Hernell O., Bläckberg L. (1994). Human milk bile salt-stimulated lipase: functional and molecular aspects. J. Pediatr..

[19] Bläckberg L., Ängquist K.A., Hemell O. (1987). Bile salt-stimulated lipase in human milk: evidence for its synthesis in the lactating mammary gland. FEBS Lett..

[20] Alvarez F., Stella V. (1989). The role of calcium ions and bile salts on the pancreatic lipase-catalyzed hydrolysis of triglyceride emulsions stabilized with lecithin. AAPS J..

[21] Christie W.W., Clapperton J.L. (1982). Structures of the triglycerides of cows' milk, fortified milks (including. infant formulae), and human milk. Int. J. Dairy Technol..

[22] Salentinig S., Phan S., Hawley A., Boyd B.J. (2015). Self-assembly structure formation during the digestion of human breast milk. Angew. Chem. Int. Ed. Engl..

[23] Folch J., Lees M., Sloane, Stanley G.H. (1957). A simple method for the isolation and purification of total lipides from animal tissues. J. Biol. Chem..

[24] Warren D.B., Anby M.U., Hawley A., Boyd B.J. (2011). Real time evolution of liquid crystalline nanostructure during the digestion of formulation lipids using synchrotron small-angle X-ray scattering. Langmuir.

[25] Williams H.D., Sassene P., Kleberg K., Bakala-N'Goma J.-C., Calderone M., Jannin V., Igonin A., Partheil A., Marchaud D., Jule E., Vertommen J., Maio M., Blundell R., Benameur H., Carrière F. (2012). Toward the establishment of standardized in vitro tests for lipid-based formulations, part 1: method parameterization and comparison of in vitro digestion profiles across a range of representative formulations. J. Pharm. Sci..

[26] Zhao P., Zhang S., Liu L., Pang X., Yang Y., Lu J., Lv J. (2018). Differences in the triacylglycerol and fatty acid compositions of human colostrum and mature milk. J. Agric. Food Chem..

[27] Kirby N.M., Mudie S.T., Hawley A.M., Cookson D.J., Mertens H.D.T., Cowieson N., Samardzic-Boban V. (2013). A low-background-intensity focusing small-angle X-ray scattering undulator beamline. J. Appl. Cryst..

[28] Holmberg K. (2002).

[29] Clulow A.J., Salim M., Hawley A., Boyd B.J. (2018). A closer look at the behaviour of milk lipids during digestion. Chem. Phys. Lipids.

[30] Agren J.J., Julkunen A., Penttilä I. (1992). Rapid separation of serum lipids for fatty acid analysis by a single aminopropyl column. J. Lipid Res..

[31] Demšar J., Curk T., Erjavec A., Gorup C., Hočevar T., Milutinovič M., Možina M., Polajnar M., Toplak M., Starič A. (2013). Orange: Data mining toolbox in python. J. Mach. Learn. Res..

[32] Pham A.C., Peng K.-Y., Salim M., Ramirez G., Hawley A., Clulow A.J., Boyd B.J. (2020). Correlating digestion-driven self-assembly in milk and infant formulas with changes in lipid composition. ACS Appl. Bio Mater..

[33] Heider M., Hause G., Mäder K. (2016). Does the commonly used pH-stat method with back titration really quantify the enzymatic digestibility of lipid drug delivery systems? A case study on solid lipid nanoparticles (SLN). Eur. J. Pharm. Biopharm..

[34] Destaillats F., de Wispelaere M., Joffre F., Golay P.-A., Hug B., Giuffrida F., Fauconnot L., Dionisi F. (2006). Authenticity of milk fat by fast analysis of triacylglycerols. J. Chromatogr. A.

[35] Hernell O. (1975). Human milk lipases. III. Physiological implications of the bile salt-stimulated lipase. Eur. J. Clin. Investig..

[36] Zangenberg N.H., Müllertz A., Kristensen H.G., Hovgaard L. (2001). A dynamic in vitro lipolysis model: I. Controlling the rate of lipolysis by continuous addition of calcium. Eur. J. Pharm. Sci..

[37] Christensen J.Ø., Schultz K., Mollgaard B., Kristensen H.G., Mullertz A. (2004). Solubilisation of poorly water-soluble drugs during in vitro lipolysis of medium- and long-chain triacylglycerols. Eur. J. Pharm. Sci..

[38] Hwang S., Lee S., Ahn I.-S., Jung J.-K. (2009). Highly efficient production of monoglycerides by the continuous removal of fatty acids from lipase-catalyzed oil hydrolysis. Biocatal. Biotransfor..

[39] Porter C.J.H., Trevaskis N.L., Charman W.N. (2007). Lipids and lipid-based formulations: optimizing the oral delivery of lipophilic drugs. Nat. Rev. Drug Discov..

[40] Murphy G.M., Signer E. (1974). Bile acid metabolism in infants and children. Gut.

[41] Encrantz J.-C., Sjövall J. (1959). On the bile acids in duodenal contents of infants and children bile acids and steroids 72. Clin. Chim. Acta.

[42] Boehm G., Braun W., Moro G., Minoli I. (1997). Bile acid concentrations in serum and duodenal aspirates of healthy preterm infants: effects of gestational and postnatal age. Neonatology.

[43] Glasgow J.F., Dinsmore H., Molla A., Macfarlane T. (1980). A comprehensive study of duodenal bile salts in newborn infants and their relationship to fat absorption. Ir. J. Med. Sci..

[44] Brockerhoff H. (1970). Substrate specificity of pancreatic lipase: influence of the structure of fatty acids on the reactivity of esters. Biochim. Biophys. Acta.

[45] Scow R.O., Desnuelle P., Verger R. (1979). Lipolysis and lipid movement in a membrane model. Action of lipoprotein lipase. J. Biol. Chem..

[46] Gittleman I.F., Pincus J.B., Saito M., Schmerzler E. (1956). Hypocalcemia occurring on the first day of life in mature and premature infants. Pediatrics.

[47] Williamson S., Finucane E., Ellis H., Gamsu H.R. (1978). Effect of heat treatment of human milk on absorption of nitrogen, fat, sodium, calcium, and phosphorus by preterm infants. Arch. Dis. Child..

[48] Kulski J.K., Hartmann P.E. (1981). Changes in human milk composition during the initiation of lactation. Immunol. Cell Biol..

[49] Sullivan S., Schanler R.J., Kim J.H., Patel A.L., Trawöger R., Kiechl-Kohlendorfer U., Chan G.M., Blanco C.L., Abrams S., Cotten C.M., Laroia N., Ehrenkranz R.A., Dudell G., Cristofalo E.A., Meier P. (2010). An exclusively human milk-based diet is associated with a lower rate of necrotizing enterocolitis than a diet of human milk and bovine milk-based products. J. Pediatr..

[50] Clulow A.J., Salim M., Hawley A., Boyd B.J. (2021). Milk mimicry – triglyceride mixtures that mimic lipid structuring during the digestion of bovine and human milk. Food Hydrocoll..

[51] Clulow A.J., Binte Abu Bakar S.Y., Salim M., Nowell C.J., Hawley A., Boyd B.J. (2021). Emulsions containing optimum cow milk fat and canola oil mixtures replicate the lipid self-assembly of human breast milk during digestion. J. Colloid Interf. Sci..

[52] de Buy Wenniger L.M., Pusl T., Beuers U. (2013). Encyclopedia of Biol. Chem..

[53] Martin J.C., Bougnoux P., Antoine J.M., Lanson M., Couet C. (1993). Triacylglycerol structure of human colostrum and mature milk. Lipids.

[54] Innis S.M. (2011). Dietary triacylglycerol structure and its role in infant nutrition. Adv. Nutr..

[55] López-López A., López-Sabter M.C., Campoy-Folgoso C., Rivero-Urgell M., Castellote-Bargalló A.I. (2002). Fatty acid and sn-2 fatty acid composition in human milk from Granada (Spain) and in infant formulas. Eur. J. Clin. Nutr..

[56] de Oliveira S.C., Bellanger A., Ménard O., Pladys P., Le Gouar Y., Dirson E., Kroell F., Dupont D., Deglaire A., Bourlieu C. (2017). Impact of human milk pasteurization on gastric digestion in preterm infants: a randomized controlled trial. Am. J. Clin. Nutr..

[57] Goldblum R.M., Dill C.W., Albrecht T.B., Alford E.S., Garza C., Goldman A.S. (1984). Rapid high-temperature treatment of human milk. J. Pediatr..

[58] Boyd B.J., Salim M., Clulow A.J., Ramirez G., Pham A.C., Hawley A. (2018). The impact of digestion is essential to the understanding of milk as a drug delivery system for poorly water soluble drugs. J. Control Release.

[59] Salim M., Khan J., Ramirez G., Murshed M., Clulow A.J., Hawley A., Ramachandruni H., Beilles S., Boyd B.J. (2019). Impact of ferroquine on the solubilization of artefenomel (OZ439) during in vitro lipolysis in milk and implications for oral combination therapy for malaria. Mol. Pharm..

[60] Binte Abu Bakar S.Y., Salim M., Clulow A.J., Hawley A., Boyd B.J. (2019). Revisiting dispersible milk-drug tablets as a solid lipid formulation in the context of digestion. Int. J. Pharm..

[61] Angelova A., Ollivon M., Campitelli A., Bourgaux C. (2003). Lipid cubic phases as stable nanochannel network structures for protein biochip development: X-ray diffraction study. Langmuir.

[62] Zabara A., Amar-Yuli I., Mezzenga R. (2011). Tuning in-meso-crystallized lysozyme polymorphism by lyotropic liquid crystal symmetry. Langmuir.

[63] Templer R.H. (1998). Thermodynamic and theoretical aspects of cubic mesophases in nature and biological amphiphiles. Curr. Opin. Colloid Interf. Sci.

[64] Meikle T.G., Drummond C., Separovic F., Conn C. (2017). Membrane-mimetic inverse bicontinuous cubic phase systems for encapsulation of peptides and proteins. Adv. Biomembr. Lipid Self-Assem..

[65] Huang Y., Gui S. (2018). Factors affecting the structure of lyotropic liquid crystals and the correlation between structure and drug diffusion. RSC Adv..

[66] Cruz-Hernandez C., Goeuriot S., Giuffrida F., Thakkar S.K., Destaillats F. (2013). Direct quantification of fatty acids in human milk by gas chromatography. J. Chromatogr. A.

