# Supplementary material for: Impact of pasteurization on the self-assembly of human milk lipids during digestion
Source: J Lipid Res. 2022 Feb 16;63(5):100183. doi: 10.1016/j.jlr.2022.100183 (PMC9065913; doi:10.1016/j.jlr.2022.100183)
Supplement: Supplemental Figures S1–S7 and Tables S1–S3 [file mmc1.docx]

**SUPPLEMENTAL INFORMATION:**

**Impact of pasteurization on the self-assembly of human milk lipids during digestion**

Syaza Y. Binte Abu Bakar^1^, Malinda Salim^1^, Andrew J. Clulow^1,2^, Adrian Hawley^2^, Donna T. Geddes^3^, Kevin R. Nicholas^1^ and Ben J. Boyd^1,4*^

^1^Drug Delivery, Disposition and Dynamics, Monash Institute of Pharmaceutical Sciences, Monash University, 381 Royal Parade, Parkville, Victoria 3052, Australia

^2^Australian Synchrotron, ANSTO, 800 Blackburn Road, Clayton, Victoria 31698, Australia

^3^School of Molecular Science, The University of Western Australia, M310, 25 Stirling Highway, Crawley Western Australia 6009, Australia

^4^Department of Pharmacy, University of Copenhagen, Universitetsparken 2, 2100 Copenhagen, Denmark

**1. Calculation to determine the concentration of each free fatty acid (FFA)**

The response factor (RF) for each fatty acid methyl esters (FAME) present in the calibration standard solution is calculated relative to the internal standard (IS) as follows:

$$RF= \frac{\% mass of FAME standard in mix \times area of IS}{\% mass of IS \times area of FAME standard in mix}$$

Based on the RF values and area under each peak of FAME using GC-FID, the concentration of each FAME was determined:

$$Concentration of FAME=\left( \frac{Adjusted area of FAME}{Area of IS}\div RF of FAME \right)\times concentration of IS$$

The concentration of each FFA was then calculated using the stoichiometric factor (Si) to convert FAME to FFA (Table S2):

**Table S1. Conversion of free fatty acid methyl ether (FAME) to free fatty acid (FFA) based on the molecular weight of FAME, their respective homogenous triglyceride (TAG) and stoichiometric factor (Si) (1).**

| **Fatty acid** | **Molecular weight of FAME (g/mol)** | **Molecular weight of TAG (g/mol)** | **Si of FAME** |
| --- | --- | --- | --- |
| C4:0 | 102.1 | 302.4 | 0.98727 |
| C6:0 | 130.2 | 386.5 | 0.98950 |
| C8:0 | 158.3 | 470.7 | 0.99116 |
| C10:0 | 186.3 | 554.9 | 0.99284 |
| C11:0 | 200.3 | 596.9 | 0.99334 |
| C12:0 | 214.4 | 639.0 | 0.99347 |
| C13:0 | 228.4 | 681.1 | 0.99402 |
| C14:0 | 242.4 | 723.2 | 0.99450 |
| C15:0 | 256.4 | 765.3 | 0.99493 |
| C16:0 | 270.5 | 807.3 | 0.99482 |
| C17:0 | 284.5 | 849.4 | 0.99520 |
| C18:0 | 298.5 | 891.5 | 0.99553 |
| C18:1 | 296.5 | 885.5 | 0.99550 |
| C18:2 | 294.5 | 879.4 | 0.99536 |
| C18:3 | 292.5 | 873.4 | 0.99533 |

$Si \left( TAG \right)= \frac{Molecular weight of TAG}{3\times molecular weight of FAME}$


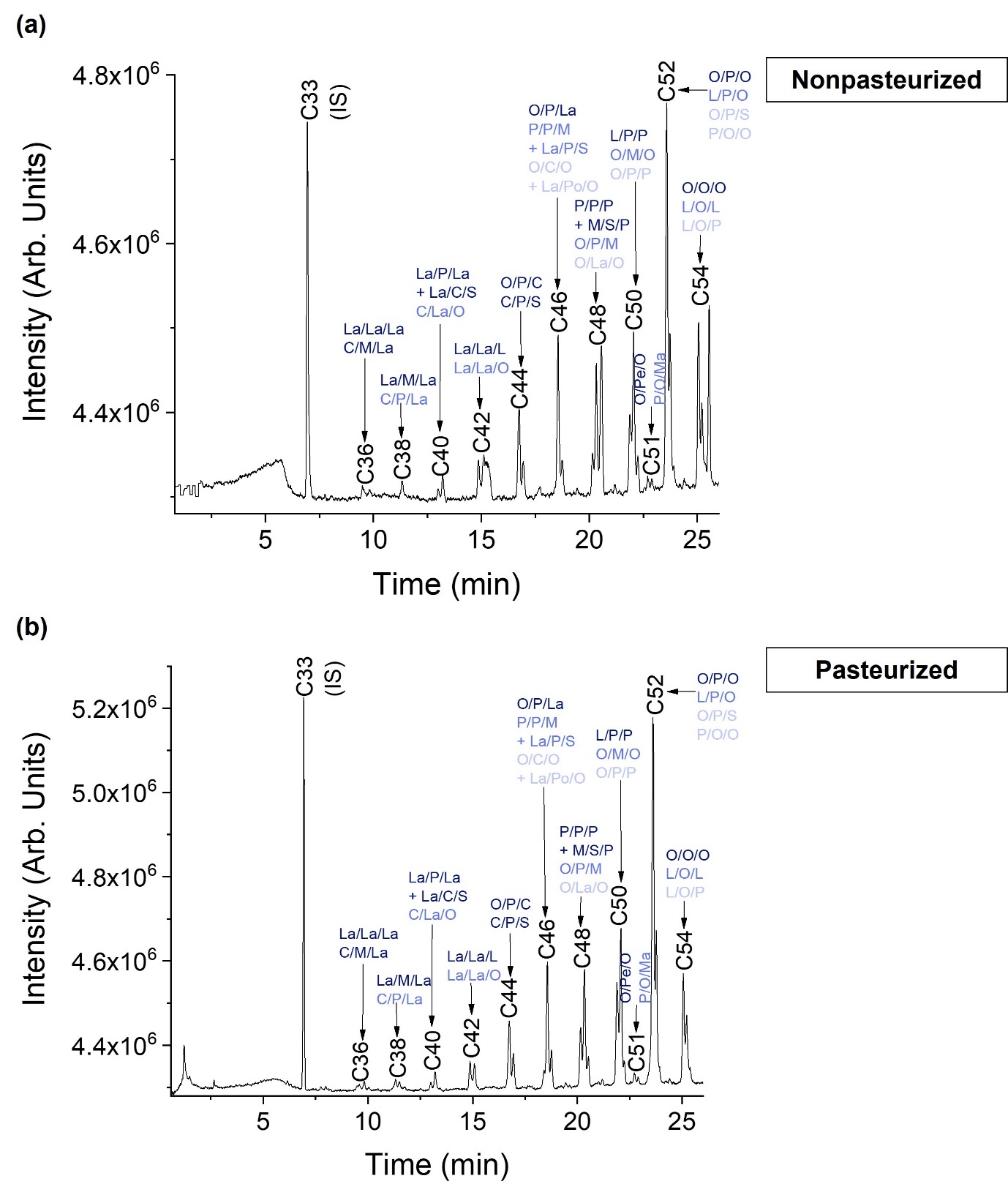


**Supplemental Figure S1. Triglyceride (TAG) composition of nonpasteurized and pasteurized human milk used in this study. Gas chromatography coupled to a flame ionisation detector (GC-FID) traces of (a) nonpasteurized and (b) pasteurized human milk lipids, dissolved in chloroform. Triundecanoin [C33 (IS)] was included as an internal standard.**


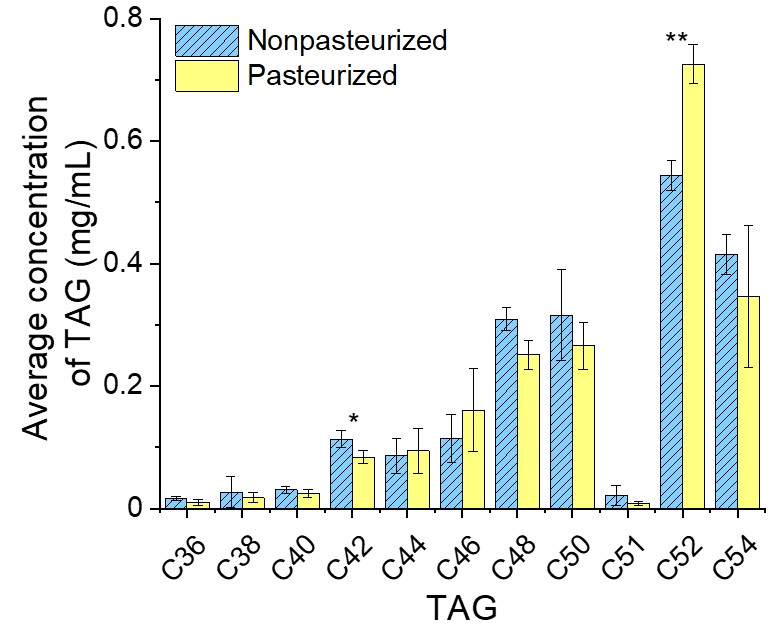


**Supplemental Figure S2. The relative amounts of TAGs in nonpasteurized and pasteurized human milk. Results are mean ± standard deviation, n = 3. * p < 0.05 and ** p < 0.01.**

**Supplemental Table S2. Average concentration and molar percentage of triglycerides (TAGs) determined from GC-FID analysis of nonpasteurized and pasteurized human milk.**

|  | | **Nonpasteurized** | | | **Pasteurized** | | |
| --- | --- | --- | --- | --- | --- | --- | --- |
| **Carbon number** | **Molecular weight (g/mol)** | **Average concentration**  **(mg/mL)** | **Molar percentage (%)** | **Contribution to overall molecular weight (g/mol)** | **Average concentration**  **(mg/mL)** | **Molar percentage (%)** | **Contribution to overall molecular**  **weight (g/mol)** |
| C36 | 639.02 | 0.0163 ± 0.0038 | 1.06 ± 0.28 | 6.75 ± 1.79 | 0.0104 ± 0.0050 | 0.68 ± 0.36 | 4.36 ± 2.28 |
| C38 | 667.07 | 0.0268 ± 0.0253 | 1.67 ± 1.60 | 11.14 ± 10.72 | 0.0175 ± 0.0080 | 1.10 ± 0.53 | 7.32 ± 3.56 |
| C40 | 695.12 | 0.0308 ± 0.0053 | 1.84 ± 0.35 | 12.76 ± 2.43 | 0.0244 ± 0.0064 | 1.47 ± 0.41 | 10.20 ± 2.88 |
| C42 | 723.18 | 0.1135 ± 0.0143 | 6.51 ± 0.85 | 47.10 ± 6.15 | 0.0841 ± 0.0107 | 4.86 ± 0.65 | 35.16 ± 4.71 |
| C44 | 751.23 | 0.0863 ± 0.0288 | 4.77 ± 1.62 | 35.80 ± 12.17 | 0.0941 ± 0.0372 | 5.23 ± 2.10 | 39.32 ± 15.77 |
| C46 | 779.29 | 0.1141 ± 0.0397 | 6.07 ± 2.14 | 47.33 ± 16.71 | 0.1606 ± 0.0677 | 8.61 ± 3.66 | 67.11 ± 28.53 |
| C48 | 807.34 | 0.3093 ± 0.0187 | 15.89 ± 0.99 | 128.32 ± 8.00 | 0.2508 ± 0.0242 | 13.00 ± 1.28 | 104.81 ± 10.37 |
| C50 | 835.39 | 0.3159 ± 0.0740 | 15.69 ± 3.71 | 131.04 ± 30.96 | 0.2659 ± 0.0387 | 13.30 ± 1.97 | 111.10 ± 16.45 |
| C51 | 848.81 | 0.0215 ± 0.0163 | 1.05 ± 0.83 | 8.94 ± 7.03 | 0.0081 ± 0.0028 | 0.40 ± 0.17 | 3.38 ± 1.42 |
| C52 | 863.45 | 0.5440 ± 0.0246 | 26.14 ± 1.21 | 225.70 ± 10.47 | 0.7259 ± 0.0320 | 35.13 ± 1.58 | 303.31 ± 13.66 |
| C54 | 891.5 | 0.4150 ± 0.0326 | 19.31 ± 1.55 | 172.18 ± 13.83 | 0.3464 ± 0.1165 | 16.24 ± 5.49 | 144.76 ± 49.00 |
|  | Total | 1.99 ± 0.28 | 100 ± 15 | 827 ± 120 | 1.98 ± 0.35 | 100 ± 18 | 831 ± 148 |

**Supplemental Table S3. Average concentration and weight percentage of free fatty acids (FFAs) determined from GC-FID analysis of digested nonpasteurized and pasteurized human milk at t = 120 min.**

|  | **Nonpasteurized** | | **Pasteurized** | |
| --- | --- | --- | --- | --- |
| **Free fatty acid** | **Average concentration**  **(mg/mL)** | **Weight percentage (*wt%*)** | **Average concentration**  **(mg/mL)** | **Weight percentage (*wt%*)** |
| C8:0 | 0.06 ± 0.02 | 0.12 ± 0.08 | 0.03 ± 0.02 | 0.09 ± 0.04 |
| C10:0 | 1.10 ± 0.14 | 2.07 ± 0.31 | 0.21 ± 0.09 | 0.70 ± 0.10 |
| C12:0 | 2.99 ± 0.33 | 5.61 ± 0.65 | 1.77 ± 0.22 | 5.88 ± 0.69 |
| C14:0 | 3.08 ± 0.43 | 5.77 ± 0.68 | 1.84 ± 0.31 | 6.11 ± 0.74 |
| C16:0 | 14.13 ± 2.85 | 26.52 ± 6.20 | 7.13 ± 1.95 | 23.72 ± 5.97 |
| C18:0 | 4.37 ± 0.58 | 8.20 ± 1.05 | 3.02 ± 1.42 | 10.05 ± 2.46 |
| C18:1 | 22.56 ± 4.82 | 42.33 ± 15.78 | 11.6 ± 2.58 | 38.75 ± 12.39 |
| C18:2 | 4.30 ± 0.57 | 8.07 ± 1.03 | 4.07 ± 1.64 | 13.55 ± 3.82 |
| C18:3 | 0.70 ± 0.02 | 1.31 ± 0.15 | 0.35 ± 0.04 | 1.17 ± 0.12 |
|  | 53.29 ± 9.76 | 100 ± 26 | 30.06 ± 8.27 | 100 ± 26 |


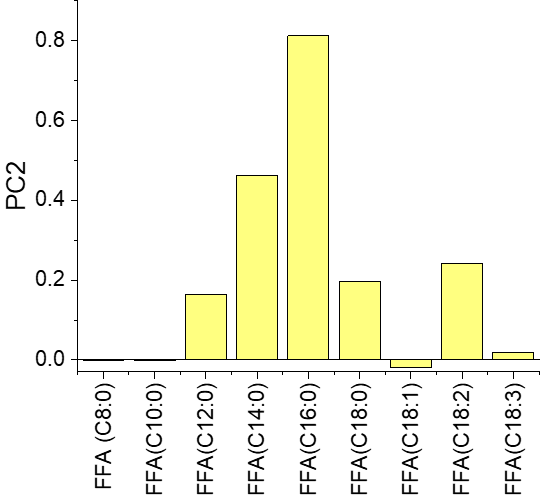


**Supplemental Figure S3. PC2 loadings plot comparing the percentages of free fatty acids (FFAs) based on the scores plot. Since PC2 accounts for 1% of the total variance, the difference between the amounts of FFAs released from each type of milk is minute.**


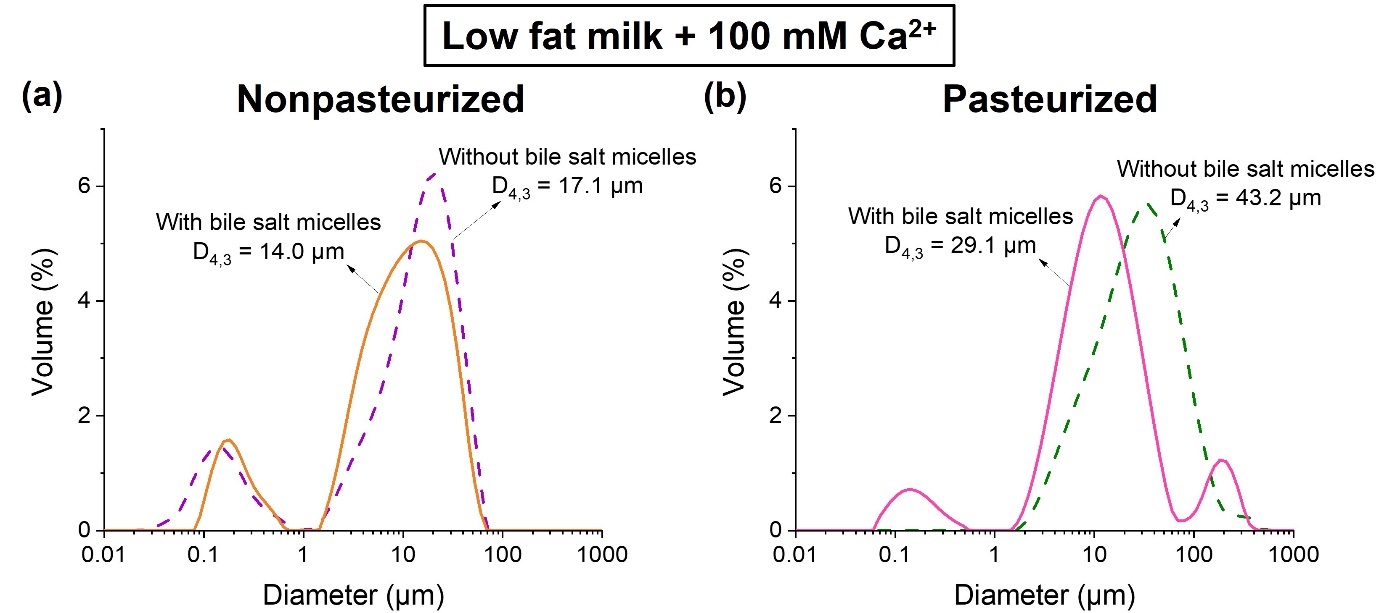


**Supplemental Figure S4. (a) Particle size distributions of digested nonpasteurized human milk (1.5% fat) without bile salt micelles (dashed purple line) and with bile salt micelles (orange line). (b) Particle size distribution of digested pasteurized human milk (1.4% fat) without bile salt micelles (dashed green line) and with bile salt micelles (pink line).**


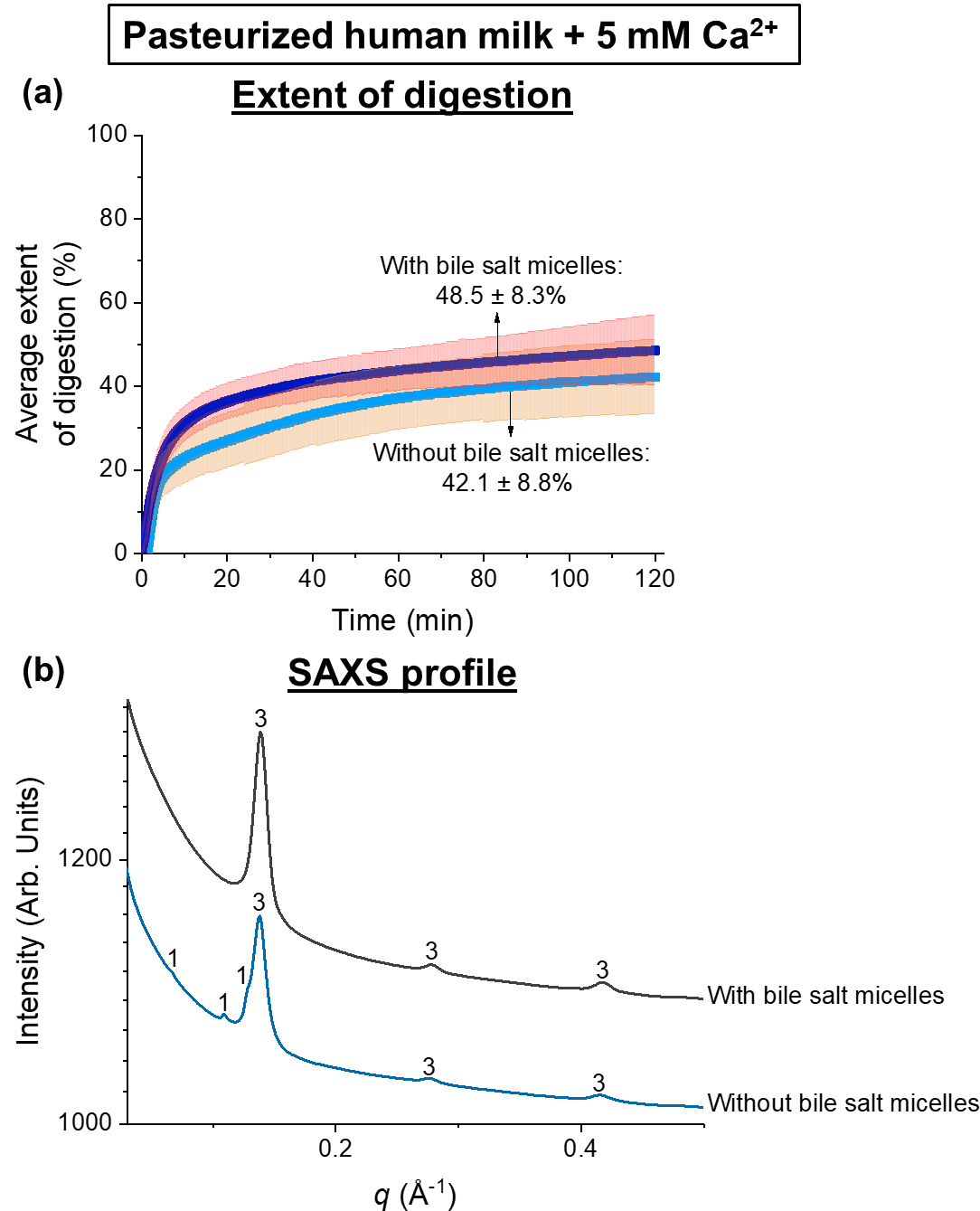


**Supplemental Figure S5. (a) Extent of digestion of high fat (5.5% fat) pasteurized human milk with 5 mM calcium in the absence (light blue with orange error bars) and presence (dark blue with red error bars) of bile salt micelles. (b) The corresponding SAXS profile where a Fd3m phase (indicated by “1”) and lamellar phase (indicated by “3”) are seen when the sample was digested without bile salt micelles (blue line). In comparison, only a lamellar phase was formed in the presence of bile salt micelles (grey line).**


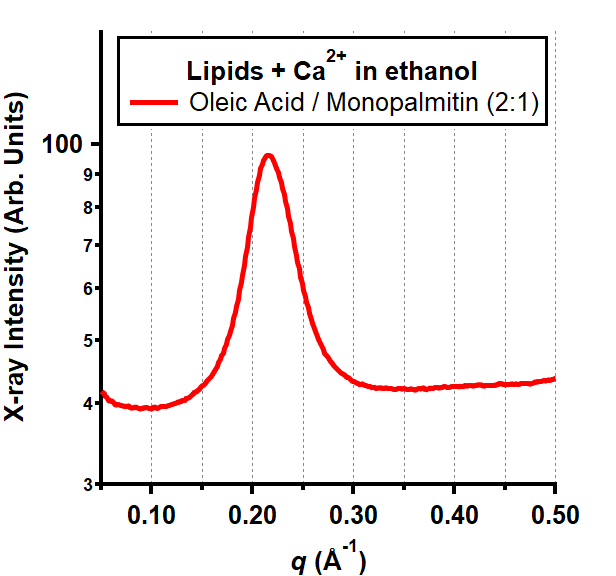


**Supplemental Figure S6. X-ray scattering profile of calcium oleate with monopalmitin present. The precipitates were formed by dissolving the lipids in the legend in ethanol saturated with calcium chloride dihydrate before one molar equivalent (with respect to oleic acid) of ethanolic sodium hydroxide was added. The scattering profiles are that of the dispersion in ethanol.**

**
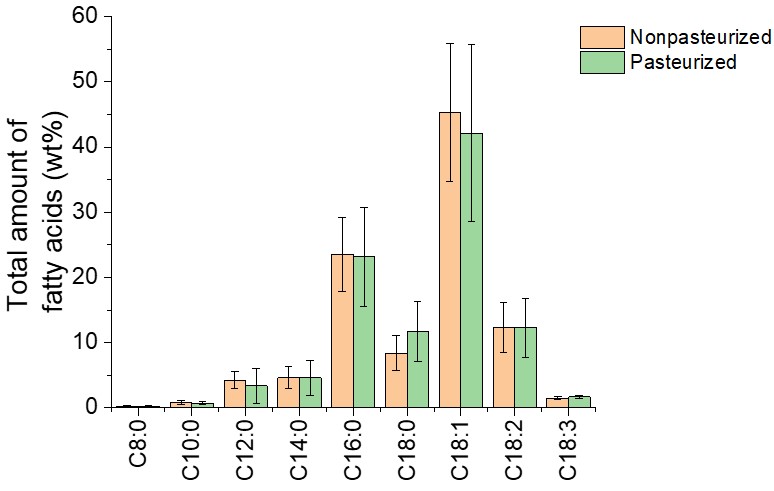
**

**Supplemental Figure S7. Total fatty acids released from undigested nonpasteurized (orange shaded bars) and pasteurized (green bars) human milk.**

**References**

1. Cruz-Hernandez, C., Goeuriot, S., Giuffrida, F., Thakkar, S. K., Destaillats, F. (2013) Direct quantification of fatty acids in human milk by gas chromatography. *J. Chromatogr. A.* 1284, 174-179.
